# Does Expert Advice Improve Educational Choice?

**DOI:** 10.1371/journal.pone.0145378

**Published:** 2015-12-21

**Authors:** Lex Borghans, Bart H. H. Golsteyn, Anders Stenberg

**Affiliations:** 1 Department of Economics and Research Centre for Education and the Labour Market (ROA), Maastricht University, Maastricht, the Netherlands; 2 Swedish Institute for Social Research (SOFI), Stockholm University, Stockholm, Sweden; TNO, NETHERLANDS

## Abstract

This paper reports evidence that an individual meeting with a study counselor at high school significantly improves the quality of choice of tertiary educational field, as self-assessed 18 months after graduation from college. To address endogeneity, we explore the variation in study counseling practices between schools as an instrumental variable (IV). Following careful scrutiny of the validity of the IV, our results indicate a significant and positive influence of study counseling on the quality of educational choice, foremost among males and those with low educated parents. The overall result is stable across a number of robustness checks.

## Introduction

The choice of a field of study at college is typically surrounded with uncertainty about the returns to education, the characteristics of occupations one can work in after graduating and the match between the individual preferences and job characteristics. A reduction in this uncertainty may provide substantial efficiency gains as an improved educational choice could enhance individuals’ job satisfaction, overall productivity and decrease study time devoted to correct initial choices. In this perspective, interesting empirical questions are if and how policy can reduce uncertainty and improve the quality of educational choices. In most OECD countries, schools employ study counselors to address this task. However, while a number of recent articles have reported that information influences educational choice, little is known if, how, and to what extent study counseling may affect educational choices. To the best of our knowledge, this study is the first to link study counseling to the quality of educational choice assessed after education has been completed and individuals have entered the labor market.

The aim of this paper is to analyze if study counseling at secondary school influences the quality of tertiary level educational choice. We use rich survey data of Dutch tertiary education graduates which include retrospective information on the use of counseling at secondary school, the name of the secondary school they graduated from, their family background, personality traits—risk-preferences, cognitive abilities, locus of control, anxiety, self-perception and self-confidence—and an assessment of the quality of their educational choice. Our main sample consists of 4,191 graduates from tertiary school. The educational system in the Netherlands is such that most individuals complete a tertiary education. According to Statistics Netherlands, around 15% end up with a diploma lower than tertiary level (http://statline.cbs.nl/statweb/). As a comparison, this exceeds the high school completion rate in the US, which peaked at around 80% in the 1970s ([1], p382). Around 18 months after tertiary school completion, the graduates in our sample are asked whether they would choose the same educational field if they had a chance to choose again. Approximately 22% of the graduates state they would have rather studied a different field of education. Table A in S2 File reveals—using a different data set—that the percentage preferring a different field in the Netherlands is relatively low compared with other countries. The relevance of this indicator is supported by its link with a higher probability of re-enrollment in education, which in turn leads to substantial efficiency losses [2].

Theoretically, we view students’ predictions of their future utility of an educational choice as noisy, such that their expected utilities partly deviate from the true future utilities associated with different educational paths. The uncertainty may regard the conditions on the labor market, the job-specific environments and the individual’s own utility function, e.g. an imprecise knowledge about own competences, motivations and/or preferences. Study counseling may reduce uncertainty in one or several respects, and thereby reduce the noise around the true values. The empirical question we raise is whether data support that an individual meeting with a counselor improves the self-assessed quality of educational choice.

A methodological challenge of our analyses is that the decision to seek help from a counselor is endogenous. Individuals who, for instance, are more uncertain (or intelligent) may seek more help from counselors and make poorer (better) choices so that conventional OLS estimates of the effect of study counseling on quality of educational choice are underestimated (overestimated). To circumvent this endogeneity problem, we explore the variation in counseling practices between schools in an instrumental variable (IV) setting. Specifically, we define our IV as the fraction of students from the same secondary school (excluding the individual him/herself) who state that they had a personal meeting with a study counselor. The variation in this variable is partly exogenous as it reflects individual counselors’ heterogeneous behaviors. Counselors typically have an independent working situation with little formal incentives to stage individual meetings with students. Our large battery of control variables can only modestly explain the incidence of seeing a counselor. This is consistent with the assumption that their behavior to a large extent is unrelated to individual or school level characteristics.

We consider the main threat to our identification strategy to be that some unobserved school specific confounders lead to an overestimation of the effects of counseling in our IV regressions. For example, if better schools generally provide more counseling, and better school environments induce a higher quality of educational choice, the effect of counseling on quality of educational choice will be overestimated. Therefore, we fully acknowledge the need to investigate if school level unobservable factors confound our IV-estimates. We perform a number of robustness checks which overall indicate little support for such “school endogeneity.” First, counseling incidence is *not* explained by the recorded school averages of parental education, school averages of immigrant status, school averages of IQ, anxiety or the other personality traits in our data set. In fact, the averages of these variables are poor predictors of counseling incidence. Second, our IV estimates remain virtually unchanged as explanatory covariates are added (the coefficient changes from -.0226 to -.0219). Third, if school endogeneity were an issue, we would expect other school specific measures of guidance policies, some of which are highly correlated with counseling incidence, to be biased by the same factors. However, using different measures of career guidance yields no significant IV estimate. In addition, while a baseline OLS model coefficient of counseling on the quality of educational choice may be biased both by individual and school endogeneity, controlling for school specific factors by adding school fixed effects has little influence on the parameter estimate. Overall, detailed checks yield results which are consistent with the key assumptions of our model with respect to school endogeneity, individual endogeneity, peer-effects and data measurement errors. Two features which should decrease the risk that the IV reflects peer effects are that our data are based on Internet surveys and include relatively few pupils per school. Also, as a robustness check, we redefine our IV, excluding students from the same school who graduated in the same year as the respondent. This does not affect our results.

Uncertainty is a classical topic in economics (e.g. [3–6]) which has developed into several branches. We wish to highlight four categories of empirical findings which are related to our study, supporting that counseling may play an important role. The first group of studies seeks to map the determinants and the extent of uncertainty about educational choice [7–10], finding that students’ knowledge about the labor market is associated with family background factors and that senior students have more accurate knowledge, implying a learning process during college years. (As sources of information, [8] reported that students primarily used newspapers and magazines (60–70%), whereas career service centers were less common (30–40%) except in the final year of college.) The second, third and fourth category of studies have focused on different parts of “the anatomy” of the uncertainty. The second group consists of a large number of studies, mainly recent, which have reported that educational choices (the choice of college major or college enrollment), educational aspirations and/or attendance rates are affected by information on objective measures, such as the expected returns to education, about own ability, about the availability of financial aid, or assistance in filling out paper work [11–21]. The third group reports that highly subjective factors may also generate uncertainty if students need to disentangle their own preferences/utility from the expectations of parents, peers, gender roles and/or other ideas about own identity [22–25]. The fourth group of studies is developed by psychologists independently of the economics literature, and shows that study counseling affects “self-efficacy”, which measures short term change in certainty about own ability and future preferences regarding individual career choice (e.g. [26–29]). In relation to these branches of the literature on educational choice, we see the incidence of counseling as a generic measure which may encompass information on objective measures (e.g. earnings) and/or address subjective issues related to uncertainty about own utility function (identity/self-efficacy). Our main analyses are agnostic with respect to the exact mechanisms, or the anatomy of the uncertainty which counselors are concerned with, but we appraise this issue via additional survey data of Dutch counselors which cover 112 of the 567 schools included in our sample.

We are aware of three articles which have evaluated study counseling practices. As outcomes, they all consider transitions from high school to college, but results have been mixed. [30] exploit the staggered roll-out of the Texas GO Center Project which targeted academically prepared students with counseling and guidance by student peers. They find college attendance rates to increase among Hispanic and low income students. [31] analyzes the impact of ten hours of individualized meetings with a professional college counselor, randomly assigned to high achieving students from relatively poor families, finding no effect on college applications but a small (statistically insignificant) effect on the quality of college chosen. [32] randomly assign college mentoring services and fee waivers for college applications to high school senior students, finding a significant impact on women’s decisions to enroll in college, but they find no significant effects for males and no effects when cash bonuses were offered without mentoring.

This paper adds to the existing literature by providing an evaluation of a widely existing policy tool, study counselors at high school, and by assessing outcomes 6–7 years after a meeting took place. We primarily address the question: does counseling influence individuals’ quality of educational choice? (Using Dutch data, there is limited scope to analyze the choice to attend college as students almost always enter tertiary education.) The assessments of educational choice are made 18 months after graduation, and thereby include individuals’ full experience of their educational choice, and their initial experience of actual (rather than expected) labor market careers. The assessment also takes into account that individuals may attach different weights to a wide array of outcomes, including non-monetary aspects, wages and job-opportunities [11]. The main finding is that counseling has a statistically significant impact on the quality of educational choice.

In terms of magnitude, one standard deviation more counseling at a school is associated with a 9 percent decrease in the probability of students preferring a different field of education in retrospect. Tentatively, based on the survey data of Dutch study counselors, we also find indications that counseling addresses uncertainty about own preferences at least as much as information about objective measures such as employment prospects. The positive effects of study counseling are strongest for males and for those whose parents have low levels of education. Overall, we consider the estimates to be large, especially since counseling is relatively inexpensive and because a low quality educational choice may be associated with substantial costs for the individual and from society’s point of view.

The plan of the paper is as follows. The next Section describes the Dutch schooling system, the data set and our key variables: counseling and the quality of the educational choice. Then, the empirical strategy is presented. After that, we show the main results, the robustness analyses, and the mechanisms. The last Section concludes.

## Dutch Schooling System, Data and Sample

In this section, we give an account of the Dutch schooling system, the sources of our data and define the sample of interest. We then present some descriptive statistics and discuss in detail the definitions and the properties of our key measures: study counseling and the quality of educational choice.

### The Dutch schooling system

The Dutch schooling system involves that at age 13, students are tracked into three different levels of secondary school. At the end of secondary school (age 16, 17 or 18), a choice has to be made regarding the field of specialization in tertiary education. A specific feature is that almost all students enroll in some form of education classified as tertiary and that only a negligible number of students start working after secondary education. The choice of field of specialization in tertiary education is important in the Dutch system since the disciplines are very specific (for instance, econometrics and economics are two separate tracks) and it is difficult to change from one specialization to another. Students in the Netherlands are allocated to three tracks when they are twelve years old. (This may attenuate the importance of counseling as the tracking could limit the possibilities for counselors to influence the quality of students’ choices.) The lowest level track at tertiary level is MBO which basically consists of learning a trade one started learning at secondary level (typical professions of graduates from this level are e.g. baker, secretary, assistant to a dentist). The next level, HBO, is also vocational but at a higher level and leads to a degree comparable to a bachelor degree (e.g. elementary and secondary school teachers, nurses, accountants, pedagogues, journalists). The highest level is university. S1 File provides an overview of the Dutch educational system and explains the abbreviations used for the different degrees. To simplify, we will refer to these tertiary levels as low, middle, and high level educational tracks.

### Data sources

We use data from a sample of Dutch graduates. Each year, the Research Centre for Education and the Labour Market (ROA) gathers information from Dutch graduates (the data are referred to as the *Schoolverlater Informatie Systeem*, abbreviated to SIS). The primary purpose of the survey is to give representative overviews of the graduates’ position on the labor market and their assessments of the quality of the education they completed.

We use information from the 2004 wave of the data. In this wave, all graduates from all levels in the Dutch educational system received a questionnaire 1.5 years after graduation. The response rate was 45 percent. Half a year after the survey took place, the respondents were approached with an additional Internet questionnaire which contains important variables for our analyses. In order to stimulate participation and deliberate answers, they were offered, upon completion of the questionnaire, a personal profile about their style to deal with choices. The survey included detailed questions on individual personality traits, such as indicators of individual discount rates, risk-preferences, cognitive ability, locus of control, anxiety, self-perception and self-confidence.

An important feature of the data set is that respondents are also asked in which secondary school they studied and in which year they graduated from this school. We use this information to construct an instrument for school counseling and measures of school averages of various characteristics. Personality traits are measured after counseling took place. If personality traits are unstable, the relationships with counseling may therefore be subject to reverse causality. [33] review the evidence on the stability of IQ and personality traits. [34] show that the rank-order trait consistency in the age group 18–22 is around 50%. The full list of the questions we used to measure personality is provided in S3 File.

Our sample of interest consists of individuals aged 20 to 30. In total, 4,191 graduates from 567 secondary schools participated. It is difficult to establish with certainty how data attrition affects estimates since, with an IV strategy, it is never possible to pin-down in detail the validity of a Local Average Treatment Effect. Nevertheless, the main impression from the attrition (see Table B in S2 File) is that the remaining observations in the second wave are similar to the first wave respondents in terms of their quality of educational choice, but constitute lower fractions of men and low level (MBO) graduates. In general, attrition implies that we overestimate the impact of counseling if students who are unaffected by counseling are underrepresented. One might suspect individuals from higher socioeconomic background to be better informed [8, 9] and have lower marginal gains from additional information. The attrition, if anything, indicates that these groups are overrepresented. The final sample contains observations from all important subgroups, but estimated results are also reported for these groups separately in the results Section.

### Measuring study counseling

To assess the occurrence of counseling, respondents were asked to consider the information they acquired in secondary school to prepare for the choice of field of tertiary education. Table 1 contains summary statistics of the respondents’ answers to the statement “I had personal conversations with the study counselor.” There are five answer categories to this statement: never (30 percent), sometimes (47 percent), regularly (16 percent), often (7 percent), very often (1 percent). Thus, about one third of the students state that they had no contact with a study counselor. The frequencies of the different answer categories appear similar for men and women, for natives and immigrants, and for those with higher and lower educated parents. (The separation between low and high education of the parents is based on the distribution of the level of education among parents. Low level indicates a level lower than the median and high level a level higher than the median level of education.) In contrast, those in the lowest secondary track are more likely to reply that they never had a personal meeting with a study counselor.

**Table 1:** Frequency of contact with study counselor.

	Never	Sometimes	Regularly	Often	Very often	Total
Women	30.3	46.6	15.6	6.3	1.1	100
Men	30.0	46.6	15.8	6.8	0.8	100
Low level secondary (VMBO)	38.4	38.3	15.2	7.0	1.1	100
Middle level secondary (HAVO)	27.8	48.0	15.5	7.6	1.1	100
High level secondary (VWO)	28.2	49.5	16.1	5.4	0.9	100
Natives	30.0	46.8	15.9	6.5	1.0	100
Immigrants	32.2	45.3	13.8	7.0	1.7	100
Parents low education	30.7	45.6	16.5	6.3	0.9	100
Parents high education	30.7	45.9	16.5	5.7	1.2	100
Total	30.2	46.6	15.7	6.5	1.0	100

Data source: Supplement survey of the 2004 SIS wave.

We construct a dummy variable which has the value 0 if a student never was in personal contact with a study counselor and 1 otherwise. Thus, we pool the answer categories “sometimes,” “regularly,” “often,” and “very often” as there may be variation in how respondents perceive these categories. Table C in S2 File shows descriptive average characteristics of individuals separated by gender and the incidence of seeing a counselor. Counseling is only associated with minor systematic differences in these variables, except that females with a higher IQ and students at the middle or high level tracks of secondary school are more likely to meet the counselor. It may be that students in these tracks better understand the importance of gathering information, and/or that counseling is offered more often as the studies are less specific and the connection to occupations is less obvious. This could make it more difficult for the students to understand the consequences of choosing a discipline.

**Fig 1:**
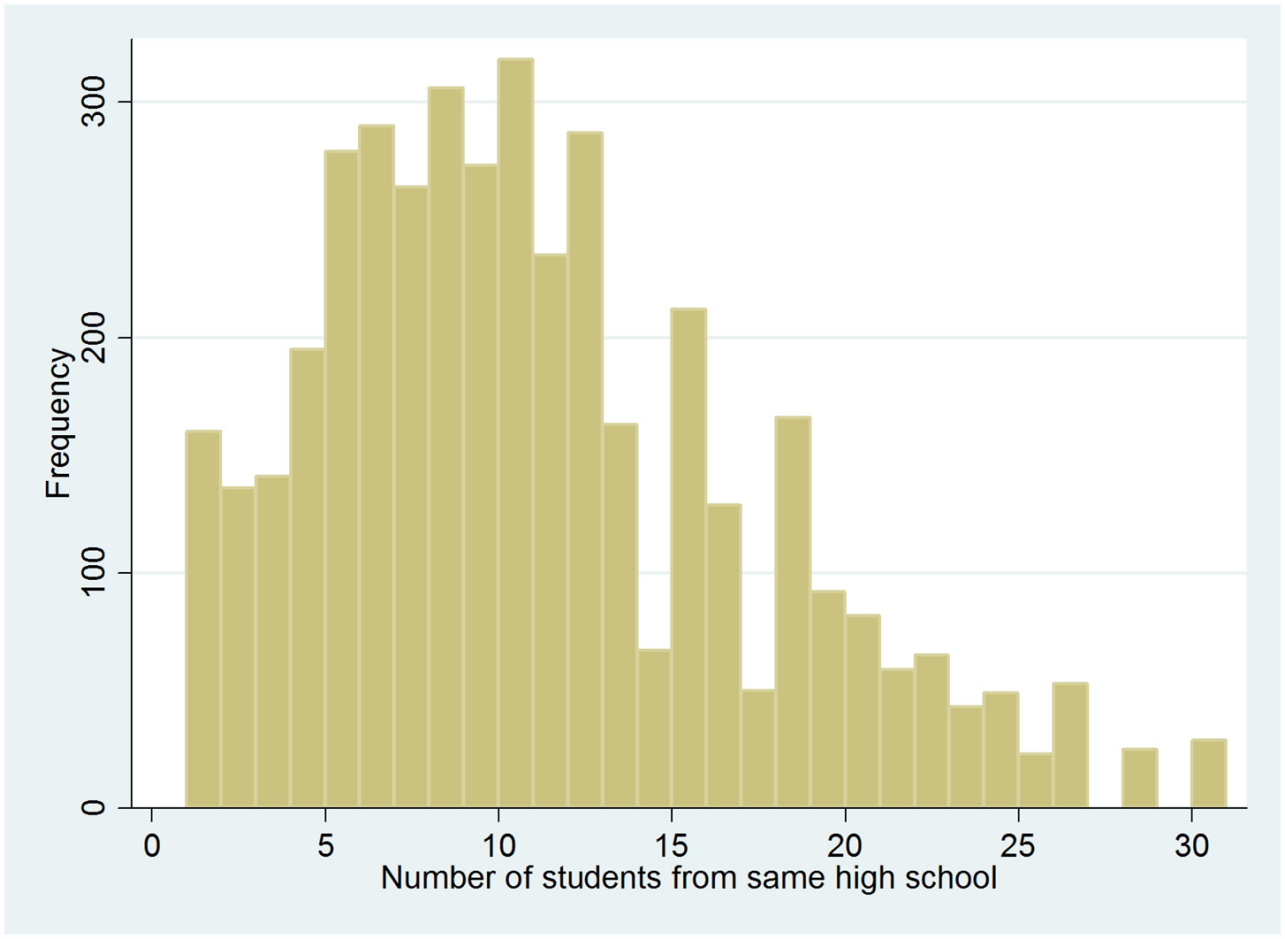
Number of students in the data set from the same secondary school. Data source: Supplement survey of the 2004 SIS wave.

**Fig 2:**
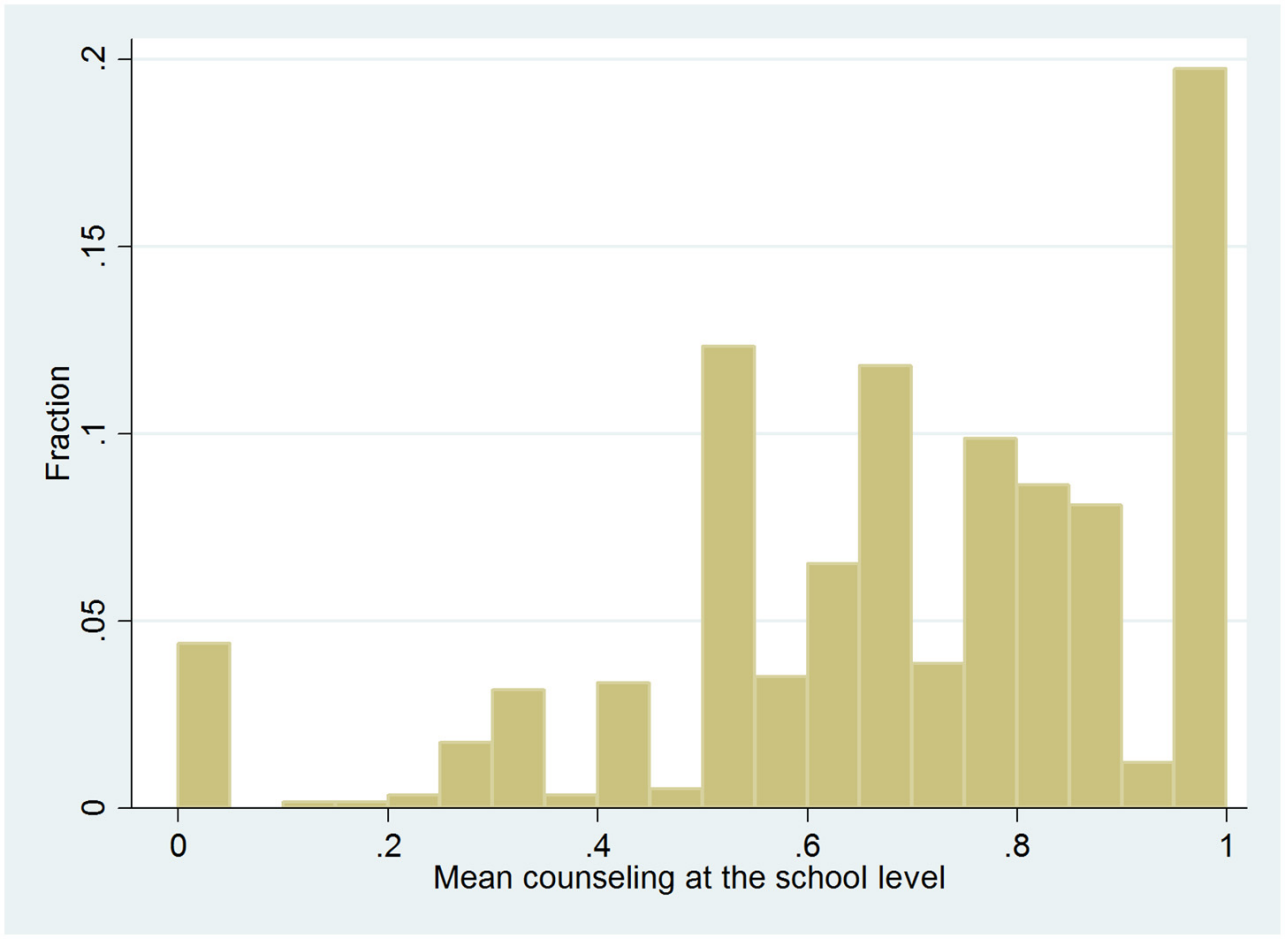
Histogram of school average amount of counseling. Data source: Supplement survey of the 2004 SIS wave. This distribution is based on school level data (so not on individual level data).

The indicator variable of individual counseling is the basis for the construction of our instrumental variable. For each individual, the IV is defined as the average counseling among students from the same secondary school, excluding the individual him/herself. (With our strategy we also avoid potential problems related to the possibility that the answers of students on questions about the quality of educational choice are correlated with the questions about counseling earlier on in the survey due to mood or personality of that student.) We assume that this variable reflects study counseling practices at secondary schools and that the variation contains an exogenous element. The credibility of this assumption is discussed in the empirical method Section and in the Section where we provide various robustness checks. The IV thus requires that each school in the sample should be represented by at least 2 respondents. Fig 1 shows the distribution of the number of students in our sample who graduated from the same school. The median is 10, the first quartile is 5 and the third quartile is 15. Fig 2 shows the average counseling frequency across the schools. Around 11% of the respondents were in a school in which every student in our sample met a counselor, while 2% of the respondents were in a school in which no respondent in our sample met a counselor. The other respondents were in schools with an average counseling between these extremes. Of the overall variance in this variable, approximately three fourths stem from between school variation and one fourth from within school variation. The within school variation may be seen as measurement error in school counseling policies, which is correlated with the number of observations we have per school. This generates heteroscedasticity in our first stage predictions which we address by allowing for a more flexible functional form (see the Section containing the robustness analyses). The observed variation in Fig 2 may not only show that individual counselors behave differently, but may also reflect a combination of school factors, students sorting into schools and randomness. The major part of this article will seek to identify and isolate the variation which is unrelated to school and student characteristics to test the hypothesis that counseling improves the quality of educational choice.

### Measuring quality of educational choice

The quality of the educational choice may be defined using a large number of criteria, to which different individuals attach different weights, e.g. the amount of leisure or commuting time, the provision of child care facilities by the employer, etc. This makes it appropriate to let the individuals themselves assess the quality of the educational choice. Our measure is an assessment 18 months after graduation, so individuals have by then attended and completed the particular educational track they chose and have had an additional 18 months to learn more about the consequences of their choice.

The question we use to assess the quality of educational choice reads “Would you in retrospect choose the same education as the one you followed again?” Answer categories are 1. “yes, same education at same college,” 2. “yes, same education but at a different school,” 3. “no, a different education,” 4. “no, I would not go and study.” We construct a dummy variable which has the value 0 if the answer was 1 or 2, and 1 if the answer was 3 or 4 (the number of graduates answering they would not go and study is negligible). The idea behind our indicator is that a person made an adequate choice if the decision based on limited information at secondary school is the same as the one stated 18 months after graduation, when consequences of the decision are known. Therefore, our outcome variable of interest can be seen as an indicator variable of low quality of the educational choice. Table 2 reveals that approximately 22% of the graduates would have chosen a different field of education, and that this is roughly equal between men and women and among people from different secondary educational tracks. Immigrants’ choices appear more often to be of low quality than natives’ choices, as is the case for students whose parents have low education compared to those with highly educated parents.

**Table 2:** Quality of educational choice.

	Prefers a different field	Prefers the same field	Total
Women	21.8	78.2	100
Men	22.3	77.7	100
Low level secondary (VMBO)	21.8	78.2	100
Middle level secondary (HAVO)	22.2	77.8	100
High level secondary (VWO)	22.0	78.0	100
Natives	21.3	78.7	100
Immigrants	28.8	71.2	100
Parents low education	22.6	77.4	100
Parents high education	20.0	80.0	100
Total	22.0	78.0	100

Data source: Supplement survey of the 2004 SIS wave. The separation between low and high education of the parents is based on the distribution of the level of education among parents. Low level indicates a level lower than the median and high level a level higher than the median level of education. Additional information using regressions is given in Table D in S2 File.

## Empirical Strategy

### Empirical model

To empirically investigate whether the quality of educational choice (*Q*_*it+1*_) may be explained by, an indicator variable for receiving counseling (*C*_*it*_), we need to take into consideration that counseling is a non-random event which potentially depends on the characteristics of the individual as well as of the school. In an OLS regression framework, this is addressed by controlling for individual characteristics *X*_*i*_ which include gender, age, secondary educational track attended, parental education, immigrant background, economic preference parameters (time and risk preference) and indicators of personality traits (locus of control, anxiety, self-perception, self-confidence, and cognitive ability), and a vector of school characteristics *X*_*j*_, containing “school pupil averages” of the same variables, where *j* denotes all individuals *j ≠ i* who attended the same secondary school as individual *i* except for individual *i* him/herself. (Time *t* should not be read as calendar year; it is merely to indicate the period prior to the assessment of educational choice.)

$$Q_{it+1}=b_{0}+b_{1}X_{i}+b_{2}X_{j}+b_{3}C_{it}+e_{i}$$ (1)

Now, as the incidence of seeing a counselor is likely to be endogenous, the error term may consist of unobserved individual characteristics *Z*_*i*_ and school specific factors *f*_*s*_:*e*_*i*_ = *b*_4_ *Z*_*i*_ + *b*_5_ *f*_*s*_ + *ε*_*i*_. If *f*_*s*_ or *Z*_*i*_ are correlated with *C*_*it*_, the parameter *b*_*3*_ will be a biased estimator of the impact of counseling on the quality of educational choice.

Table 3 shows results from the baseline OLS regression, indicating a beneficial but small association of study counseling with the quality of the educational choice. The coefficient shows that attending a counselor is negatively associated with the probability to make a low quality educational choice. This estimate is potentially biased due to individual characteristics *Z*_*i*_, school specific factors *f*_*s*_, or both. The school endogeneity implies that schools’ provision of counseling is correlated with their pupils’ abilities to gather information. Thus, even if counseling has no effect on individuals’ choices, there may be a spurious correlation between counseling and quality of educational choice.

**Table 3:** OLS estimates of the relationship between study counseling and quality of the educational choice.

	(1)	(2)	(3)	(4)	(5)
	Prefers a different field	Prefers a different field	Prefers a different field	Prefers a different field	Prefers a different field
Counseling	-0.0020*	-0.0019*	-0.0020*	-0.0020*	-0.0017
	(0.0011)	(0.0011)	(0.0011)	(0.0011)	(0.0011)
Men (average)		-0.0150		-0.0187	-0.0171
		(0.0398)		(0.0424)	(0.0424)
Age (average)		-0.0046		-0.0041	-0.0024
		(0.0071)		(0.0078)	(0.0078)
Educ father (average)		0.0086		0.0096	0.0115
		(0.0100)		(0.0108)	(0.0108)
Educ mother (average)		-0.0065		-0.0018	-0.0018
		(0.0100)		(0.0107)	(0.0107)
Immigrant (average)		0.1460***		0.0760	0.0678
		(0.0520)		(0.0570)	(0.0569)
Discount rate (average)		0.0004		0.0005	0.0004
		(0.0008)		(0.0009)	(0.0009)
Risk preference (average)		-0.0002		-0.0005	-0.0005
		(0.0005)		(0.0006)	(0.0006)
Locus of control (average)		0.0148		0.0288	0.0264
		(0.0222)		(0.0235)	(0.0235)
Anxiety (average)		-0.0459**		-0.0269	-0.0264
		(0.0198)		(0.0210)	(0.0210)
Self-perception (average)		-0.0748***		-0.0331	-0.0355
		(0.0230)		(0.0245)	(0.0245)
Self-confidence (average)		-0.0261		-0.0115	-0.0100
		(0.0220)		(0.0233)	(0.0233)
Cognitive ability (average)		-0.0044		-0.0073	-0.0090
		(0.0082)		(0.0089)	(0.0089)
Male			0.0128	0.0153	0.0136
			(0.0143)	(0.0153)	(0.0153)
Age = 21			-0.1286***	-0.1288***	-0.1247***
			(0.0417)	(0.0418)	(0.0417)
Age = 22			-0.1666***	-0.1632***	-0.1578***
			(0.0395)	(0.0398)	(0.0398)
Age = 23			-0.1398***	-0.1375***	-0.1349***
			(0.0389)	(0.0394)	(0.0393)
Age = 24			-0.1387***	-0.1370***	-0.1344***
			(0.0389)	(0.0396)	(0.0395)
Age = 25			-0.1057***	-0.1028**	-0.1017**
			(0.0399)	(0.0409)	(0.0408)
Age = 26			-0.1136***	-0.1129***	-0.1152***
			(0.0415)	(0.0428)	(0.0427)
Age = 27			-0.1519***	-0.1479***	-0.1481***
			(0.0441)	(0.0455)	(0.0454)
Age = 28			-0.1454***	-0.1403***	-0.1351***
			(0.0492)	(0.0509)	(0.0508)
Age = 29			-0.1277**	-0.1250**	-0.1255**
			(0.0576)	(0.0595)	(0.0594)
Age = 30			-0.2700***	-0.2668***	-0.2669***
			(0.0664)	(0.0680)	(0.0678)
Educ father			-0.0005	-0.0017	-0.0026
			(0.0038)	(0.0041)	(0.0041)
Educ mother			-0.0049	-0.0048	-0.0043
			(0.0037)	(0.0040)	(0.0040)
Middle level sec. school			0.0428**	0.0436**	0.0372**
			(0.0177)	(0.0178)	(0.0178)
High level sec. school			0.0338*	0.0371*	0.0331*
			(0.0196)	(0.0199)	(0.0198)
Immigrant			0.0746***	0.0586**	0.0582**
			(0.0215)	(0.0238)	(0.0238)
Discount rate			-0.0000	-0.0001	-0.0001
			(0.0003)	(0.0003)	(0.0011)
Risk preference			0.0002	0.0003	0.0013**
			(0.0002)	(0.0002)	(0.0006)
Locus of control			-0.0090	-0.0128	-0.0421**
			(0.0078)	(0.0083)	(0.0190)
Anxiety			-0.0203***	-0.0170**	-0.0192
			(0.0069)	(0.0074)	(0.0362)
Self-perception			-0.0459***	-0.0413***	-0.0285**
			(0.0082)	(0.0088)	(0.0119)
Self-confidence			-0.0149*	-0.0141*	0.0036
			(0.0077)	(0.0082)	(0.0111)
Cognitive ability			-0.0016	-0.0004	0.0129
			(0.0033)	(0.0035)	(0.0097)
Discount rate squared					-0.0000
					(0.0000)
Risk preference squared					-0.0000*
					(0.0000)
Locus of control squared					0.0101*
					(0.0060)
Anxiety squared					0.0004
					(0.0044)
Self-perception squared					0.0093*
					(0.0051)
Self-confidence squared					0.0109**
					(0.0044)
Cognitive ability squared					-0.0019
					(0.0013)
Constant	0.2205***	0.4264**	0.3785***	0.5192**	0.4622**
	(0.0064)	(0.1988)	(0.0518)	(0.2107)	(0.2235)
Observations	4,191	4,191	4,191	4,191	4,191
R-squared	0.001	0.009	0.029	0.032	0.038

Notes: Standard errors in parentheses,

*** p<0.01,

** p<0.05,

* p<0.1.

Data source: Supplement survey of the 2004 SIS wave. The dependent variable is a dummy variable (0 = does not prefer a different field of education in retrospect, 1 = prefers a different field of education in retrospect). Counseling is standardized at the school level as described in the data section. Educ Father and Mother represent the highest level of education that the father or mother graduated from. “average” indicates that school averages have been calculated.

The individual endogeneity (*Z*_*i*_) may be thought of in terms of uncertainty about future career choice. Students who are more uncertain may be more likely to seek counseling, but may also be more likely to end up with a low quality educational choice (cf. seeing a medical doctor increases the probability of being sick). This would make the OLS coefficient underestimate the impact of counseling. Of course, it may also be that students who are better at gathering information are more likely to see a counselor, leading to a reverse bias.

To address the individual endogeneity, we employ an instrumental variable strategy. The idea originates from a widespread view among professional study counselors that there is considerable heterogeneity in counseling activity between high schools which stems from the individual counselor(s) who may either be very active or offer counseling of such quality that they attract students’ visits. To the extent that this variation is uncorrelated with school specific characteristics and/or individual traits of the pupils, it will generate an exogenous variation which may be explored as an instrumental variable (IV) to explain the incidence of seeing a counselor. We employ as IV the average frequency of counseling among students *j ≠ i* from the same school as the respondent, *S*_*jt*_, to proxy for the counseling practices at the school. *S*_*jt*_ does not include the individual’s own endogenous decision, but is assumed to predict his/her probability of receiving counseling. In a second stage regression, the predicted value (*Ĉ*_*it*_) is used as an explanatory variable for *Q*_*it+1*_. Formally, the following model is estimated:

$$C_{it}=\alpha_{0}+\alpha_{1}X_{i}+\alpha_{2}X_{j}+\alpha_{3}S_{jt}+\varepsilon_{i}$$ (2)

$$Q_{it+1}=\beta_{0}+\beta_{1}X_{i}+\beta_{2}X_{j}+\beta_{3}{\hat{C}}_{it}+\upsilon_{i},$$ (3)

in which *ε*_*i*_ and *υ*_*i*_ are error terms in the respective regressions and the *α* and *β* parameters are to be estimated. The second stage estimate of _*3*_ is the parameter of main interest. It reflects the Local Average Treatment Effect (LATE) and is only valid for those who are affected by an increase in study counseling activity [35]. To obtain the average treatment effect of the whole population, one would require that our IV affects the behavior of all individuals in the same way. In an effort to find out which individual characteristics are associated with our LATE estimates, we estimated *C*_*it*_ = *λ*_*0*_ *+ λ*_*1*_ *S*_*jt*_ *+ λ*_*2*_ *X*_*it*_ *+ λ*_*3*_ *(S*_*jt*_ )*X_*it*_. The coefficients of the interaction variables *λ*_*3*_ could then be informative, but none of them are significant. For the subsample of natives, the interaction between our IV and the discount rate is positive and significant, suggesting that sensitivity to counselor’s behavior depends on the discount rate. The result holds for the subsample of males but not for females. For immigrants, we find that those with an internal locus of control are affected significantly more than immigrants with an external locus of control. Complete results are available on request.

In theory, one may expect that the individuals most affected by the counseling activity at the school would be those who tend to have less accurate information at the outset, e.g. with immigrant backgrounds or with parents who have low educational attainments. Uncertainty about the own utility function may strengthen or weaken this tendency, depending on how expectations of parents, peers, gender roles and/or own identity vary across socioeconomic groups and whether they generate certainty (e.g. “I want to do what my mother/father does”) or uncertainty (e.g. a conflict between complying with others’ expectations and pursuing a different educational path). Counselors could make a difference either by encouraging individuals to challenge these expectations or by strengthening the preferences generated by these expectations.

### Validity of our instrumental variable

The validity of our empirical strategy hinges on that (1) the IV is able to predict that individuals seek help from a counselor but (2) is uncorrelated, or unconfounded, with potential unobservable variables which simultaneously influence the probability of seeing a counselor and the outcome variable *Q*_*it+1*_. We exploit that advisors work independently, without strong incentives to improve the student’s educational choice. This may generate a random element to the counselor's activity at a given school. The underlying assumptions of the unconfoundedness condition are not directly testable, but below we address their credibility by discussing measurement issues, school specific confounders, and individual confounders. [36] emphasize the importance of a well-developed theoretical “story.” [37] (p6) makes the following remark: “…minimal identifying assumptions must be justified or rationalized on the basis of a priori argument, outside evidence, intuition, theory, or some other means outside the model. While the necessity to make these types of arguments may at first seem dismaying, it can also be argued that they are what social science is all about, which is using one’s comprehensive knowledge of society to formulate theories of how social forces work, to make informed judgments about those theories, and debating with other social scientists what the most supportable assumptions are.” First stage regressions, presented in Table 4, indicate that students are much more likely to see a study counselor if they attended schools where counseling of other individuals was more frequent. Thus, the first condition of our IV strategy appears to hold (*F*-statistic of 40.3), even after including a large number of control variables.

**Table 4:** First stage results: the effect of average amount of counseling by students of the same secondary school on individual’s counseling.

	(1)	(2)	(3)	(4)	(5)
	Counseling	Counseling	Counseling	Counseling	Counseling
Instrument	0.6289***	0.5916***	0.6116***	0.5792***	0.5651***
(average counseling)	(0.0904)	(0.0913)	(0.0905)	(0.0912)	(0.0912)
Men (average)		-0.1680		0.0380	0.0050
		(0.5642)		(0.6044)	(0.6038)
Age (average)		-0.0643		-0.1814	-0.1882*
		(0.1000)		(0.1114)	(0.1114)
Educ father (average)		0.0424		0.0105	-0.0094
		(0.1422)		(0.1533)	(0.1533)
Educ mother (average)		-0.2698*		-0.2775*	-0.2703*
		(0.1411)		(0.1522)	(0.1521)
Immigrant (average)		-0.4950		-0.4711	-0.4151
		(0.7376)		(0.8125)	(0.8122)
Discount rate (average)		-0.0115		-0.0061	-0.0046
		(0.0119)		(0.0128)	(0.0128)
Risk preference (average)		-0.0080		-0.0110	-0.0110
		(0.0076)		(0.0080)	(0.0080)
Locus of control (average)		-0.2148		-0.1951	-0.1812
		(0.3138)		(0.3352)	(0.3351)
Anxiety (average)		-0.0916		-0.1530	-0.1458
		(0.2808)		(0.2993)	(0.2990)
Self-perception (average)		0.2014		0.3002	0.2951
		(0.3254)		(0.3485)	(0.3485)
Self-confidence (average)		-0.2703		-0.4128	-0.4187
		(0.3115)		(0.3322)	(0.3320)
Cognitive ability (average)		0.0914		0.0091	0.0217
		(0.1157)		(0.1265)	(0.1265)
Male			0.1144	0.1011	0.0998
			(0.2035)	(0.2173)	(0.2173)
Age = 21			0.0450	0.0800	0.0768
			(0.5942)	(0.5953)	(0.5948)
Age = 22			0.0348	0.1097	0.0447
			(0.5630)	(0.5669)	(0.5666)
Age = 23			0.0877	0.2195	0.1781
			(0.5547)	(0.5612)	(0.5607)
Age = 24			-0.3170	-0.1394	-0.1789
			(0.5543)	(0.5641)	(0.5635)
Age = 25			-0.0901	0.1411	0.1540
			(0.5684)	(0.5826)	(0.5819)
Age = 26			-0.1729	0.1364	0.1708
			(0.5915)	(0.6091)	(0.6084)
Age = 27			-0.0729	0.2031	0.1846
			(0.6278)	(0.6473)	(0.6468)
Age = 28			-0.7189	-0.3886	-0.4526
			(0.7009)	(0.7245)	(0.7241)
Age = 29			-1.5969*	-1.1337	-1.1336
			(0.8202)	(0.8473)	(0.8461)
Age = 30			0.1753	0.6233	0.6340
			(0.9450)	(0.9679)	(0.9668)
Educ father			-0.0089	-0.0019	0.0074
			(0.0536)	(0.0579)	(0.0579)
Educ mother			-0.0123	0.0360	0.0294
			(0.0531)	(0.0575)	(0.0575)
Middle level sec. school			1.2358***	1.2573***	1.2883***
			(0.2511)	(0.2521)	(0.2530)
High level sec. school			1.3081***	1.3855***	1.3970***
			(0.2778)	(0.2820)	(0.2820)
Immigrant			-0.1604	-0.0270	-0.0219
			(0.3061)	(0.3391)	(0.3390)
Discount rate			-0.0035	-0.0027	-0.0170
			(0.0044)	(0.0047)	(0.0157)
Risk preference			0.0006	0.0020	0.0005
			(0.0026)	(0.0027)	(0.0084)
Locus of control			-0.0296	0.0067	0.0439
			(0.1105)	(0.1184)	(0.2712)
Anxiety			0.0764	0.0937	1.3762***
			(0.0988)	(0.1055)	(0.5149)
Self-perception			-0.0486	-0.0936	-0.2611
			(0.1172)	(0.1257)	(0.1698)
Self-confidence			0.0670	0.1179	-0.0288
			(0.1092)	(0.1167)	(0.1577)
Cognitive ability			-0.0216	-0.0242	-0.0324
			(0.0467)	(0.0500)	(0.1376)
Discount rate squared					0.0002
					(0.0002)
Risk preference squared					0.0000
					(0.0001)
Locus of control squared					-0.0066
					(0.0861)
Anxiety squared					-0.1590**
					(0.0621)
Self-perception squared					-0.1164
					(0.0726)
Self-confidence squared					-0.0840
					(0.0632)
Cognitive ability squared					0.0007
					(0.0181)
Constant	0.1198	3.8569	-0.8636	5.6306*	3.6021
	(0.0903)	(2.8154)	(0.7373)	(3.0001)	(3.1859)
Observations	4,191	4,191	4,191	4,191	4,191
R-squared	0.011	0.014	0.022	0.025	0.030

Notes: Standard errors in parentheses,

*** p<0.01,

** p<0.05,

* p<0.1.

Data source: Supplement survey of the 2004 SIS wave. The dependent variable “Counseling” is standardized at the school level as described in the data section. Educ Father and Mother represent the highest level of education that the father or mother graduated from. “average” indicates that school averages have been calculated “Average counseling in same secondary school” is the average amount of counseling by students of the same secondary school as the respondent, standardized to mean zero and standard deviation 1. Educ Father and Mother represent the highest level of education that the father or mother graduated from. “average” indicates that school averages have been calculated.

#### Measurement issues

In this subsection, we consider the accuracy of the collected data on personality traits and whether our IV really captures the counseling activity at the schools. We also give an account of a correction in the standardization of the counseling variable.

Looking at our first stage regression results, a reservation one might have is that except for the IV, the level of educational track and the individual level of anxiety, the covariates generally do not significantly explain the occurrence of counseling. This may indicate that the personality traits are poorly measured. We therefore ran a regression with the level of tracking as the dependent variable, which we would expect to be highly endogenous and correlated with these variables. We find that the covariates are significant and in the expected direction (e.g. the IQ variable is associated with a *t*-value of 23.7). Another interpretation of the covariates’ lack of explanatory power is that counseling incidence may have a large random component. If counseling would be truly random, the OLS estimate would have a causal interpretation. However, since unobserved confounders may not have been included as covariates, we cannot be certain it is truly random. Therefore, it is important to explore the relationship using an IV technique.

Another concern is that our IV may mismeasure the true counseling activity of the full student population at schools. To examine this issue, we link our data to an additional survey data set from 2008, where study counselors from middle and high level Dutch high schools were approached to fill out a questionnaire about their activities and to what extent students in their schools sought help from the study counselors. Using the school name which was available in both data sets, we merged the information from this counselor data set with our sample (1168 students from 134 schools). The data indicate that our IV indeed does pick up school study counseling practices. In Table 5, answers are shown for counselors from schools where our IV is above and below median respectively. The survey answers of the counselors in schools with above median IV, compared with below median IV, indicate (1) that there were more counselors active, (2) that the percentage of the students seeking individual study counseling was higher, and (3) that counselors more often stated there was enough information available in the school to prepare students for their choice. These differences are significant at the 1 percent level.

**Table 5:** Information from additional survey among study counselors in 2008.

	Group with IV below median	Group with IV above median
Mean number of counselors at middle level secondary school	0.663	0.850
	(0.037)	(0.039)
Mean number of counselors at high level secondary school	0.551	0.817
	(0.028)	(0.039)
Percentage middle level secondary school students who attends a counseling session once	38.95	47.03
	(1.64)	(1.62)
Percentage middle level secondary school students who attends a counseling session more than once	7.87	14.33
	(0.55)	(1.00)
Percentage high level secondary school students who attends a counseling session once	61.51	66.93
	(1.58)	(1.41)
Percentage high level secondary school students who attends a counseling session more than once	22.98	27.74
	(1.21)	(1.34)
Mean answer to “does school have enough information to guide students”	0.780	0.825
	(0.018)	(0.016)

Notes: standard errors are reported in parentheses.

Given that our IV captures the behavior of the individual counselors at schools, a high frequency of visits may indicate that they are very active, or that they provide counseling of high quality which attracts students to come and visit them. This implies that the frequency and the quality of counseling plausibly correlate, and it would make us at least partly evaluate the effects of high quality counseling rather than just the average quality of counseling.

A final measurement issue is that we standardize the counseling variable using the distribution of average counseling at the level of the secondary school. This requires a consistent measure of the variance, but our average counseling at the school level is likely to contain measurement error which is inversely related to the number of observed students per school. We then risk overestimating the variance and thereby also the potential impact of a standard deviation change in the intensity of study counseling. We correct for this by running a regression of the measured variance at the school level on a constant and the inverse of the number of students per school. The constant of this regression gives a consistent measure of the variance corrected for measurement error. Formally, we assume our approximate school specific probability of seeing a counselor in a school $\left({\bar{s}}_{jt}\right)$ is equal to the true school average $\left({\bar{s}}_{sch}\right)$ plus measurement error (*e*_*sch*_), with the error inversely related to the observed number of students per school (*N*_*sch*_). Then, $Var\left({\bar{s}}_{jt}\right)=Var\left({\bar{s}}_{sch}\right)+Var\left(e_{sch}\right)$, where $Var\left(e_{sch}\right)=\frac{{\bar{s}}_{sch}\left(1-{\bar{s}}_{sch}\right)}{N_{sch}}$. The variance corrected for measurement error is the constant (γ_0_) in the regression: $Var\left({\bar{s}}_{jt}\right)=\gamma_{0}+\gamma_{1}\left(1/N_{sch}\right)+\varepsilon_{sch}$.

#### School specific confounders

The main threat to our identification strategy is arguably that unobserved school specific characteristics are related to study counseling practices. For instance, schools from relatively rich areas may tend to provide better or more counseling, but their students may be good at gathering information in the first place. To address this, *X*_*j*_ in Eq (3) includes a large set of school average characteristics: parental education, immigrant status, time preference, risk aversion, cognitive ability, anxiety, self-perception, self-confidence and locus of control. The robustness of our results to the inclusion of these control variables serves as a first indication that school specific factors do not undermine our IV estimates.

In addition, one may note that if our IV reflects some unobserved school quality variable, one would also expect the other school specific average characteristics to explain counseling incidence. However, none of 12 parameters pertaining to the school averages of parents’ social background and/or pupils’ personality traits is significant at a five percent level. In contrast, our IV which is also constructed as the average of students *j ≠ i* from the same school as the respondent, is highly significant with *p*-values below .001. The results are thus consistent with the idea that the probability of seeing a counselor contains a non-trivial element of random variation across schools.

However, since it is a key factor of this study, let us for the sake of argument assume that school endogeneity tends to exaggerate the impact of counseling. The baseline OLS estimates in Table 3, which are close to zero, would then make sense only if some other unobservable also generates bias towards zero (e.g. uncertain individuals tend to see counselors). If we add school fixed effects, school endogeneity is taken into account (while individual endogeneity within school remains). This specification yields a *b*_*3*_ coefficient of -.0011 (*p*-value .374), which is similar to Table 3. Thus, when controlling for unobserved school level factors, there is only a small impact on the estimate, suggesting that potential bias originating from school endogeneity is modest (see the robustness analysis Section for fixed effects in the IV framework). The coefficient could be driven towards zero by measurement error bias, which is exacerbated when one uses fixed effects. However, with the stated assumptions, one would have expected bias from individual endogeneity to generate a positive coefficient (underestimating the effect of counseling) in Table 3.

Outside of school hours, one might also suspect that families’ support differs systematically between schools. In our data, individuals were asked whether they formed an image of their education or profession via family members’ education or profession. About 30% of the pupils stated that they formed the image using such information, but the school average of this variable is unrelated to the individual’s use of this information (i.e. the first stage is insignificant). Support from the family can therefore not account for school specific variation in the amount of help students receive when making their choice.

Still, since an IV approach does not allow us to technically exclude the possibility that a confounder exists which is school specific, correlated with *S*_*jt*_ and *Q*_*it+1*_, but uncorrelated with the control variables, we also ran IV regressions using other school specific measures of actions to guide students in their educational choices. These measures include “lessons about educational choice were provided” (82 percent stated there were), whether “people came to talk about their professions” (52 percent) and “how often did you go to an information day?” (5 percent reported zero, 10 percent five times or more, the mode is two). These are all positively correlated with the counseling indicator (significant at a .01 level) but yield no statistically significant IV estimates. Thus, potential school specific confounders must, in addition to the conditions above, be uncorrelated with these other school specific guidance policies (further discussed in the Section which shows the robustness analyses).

#### Individual confounders

Concerning unobserved individual traits, the IV is not based on any direct information on individual *i*. Potential bias may then only arise indirectly, through correlations between individual traits and school quality (which we just discussed) or peer-effects, which might be considered a hybrid between individual and school specific traits. Peer effects originate from the social environments generated among members of a group of friends or of a classroom. This is a problem if peers *j* affect individual *i* but also if student *i* affects peers (and who potentially in turn will influence him/her and so on, the so called reflection problem [38]).

The main concern here is that individuals from the same school in our survey met the same classroom/teacher, or were in the same circle of friends. Our IV could then pick up e.g. that forward looking peers affect both the probability of seeking counseling and the quality of educational choice, which would bias our estimates.

Our sample consists of relatively few respondents from each school, who each completed an Internet survey. For peers to have a major influence on both the probability of seeing a counselor and the quality of educational choice, one needs to assume (1) that the relatively few respondents from each school were part of the same circle of friends/classrooms or other partial environments when in high school; (2) that they influence one another to complete the Internet survey; (3) that our respondents still were in contact with their high-school peers 18 months after graduation from tertiary education; (4) that few others from the same school, outside the peer group, completed the survey (as they would dilute the peer effect on our IV); and (5) that assumptions 1–4 would have to hold across a non-trivial proportion of our 567 schools represented. To us, the chance that these requirements are all fulfilled appears too improbable to be of major importance, especially given the fact that peer-effects are partly included in our average school characteristics, which we found to have modest influence in our regressions. To decrease the risk of peer effects even further, we redefined the IV in robustness checks to reflect the average incidence of counseling for students from the same school but who did not graduate in the same year as the respondent. We report results from these IV regressions in the robustness analyses Section.

## Results

Our main results—presented in Table 6—indicate that in a school which offers one standard deviation more counseling, the probability to prefer a different field of education is reduced by approximately 2 percentage points. The estimate remains robust as we gradually include background characteristics and personality traits of individuals and same school pupils’ averages. The statistical power drops slightly between columns (4) and (5) from a *p*-value of .049 to .063 when we add the squares of the personality traits.

**Table 6:** The effect of counseling on the quality of educational choice.

	(1)	(2)	(3)	(4)	(5)
	Prefers a different field	Prefers a different field	Prefers a different field	Prefers a different field	Prefers a different field
Counseling	-0.0226**	-0.0227**	-0.0225**	-0.0228**	-0.0219*
	(0.0106)	(0.0114)	(0.0108)	(0.0115)	(0.0118)
Men (average)		-0.0198		-0.0189	-0.0179
		(0.0416)		(0.0443)	(0.0441)
Age (average)		-0.0060		-0.0080	-0.0062
		(0.0074)		(0.0084)	(0.0084)
Educ father (average)		0.0094		0.0098	0.0112
		(0.0105)		(0.0112)	(0.0112)
Educ mother (average)		-0.0133		-0.0086	-0.0083
		(0.0110)		(0.0118)	(0.0117)
Immigrant (average)		0.1285**		0.0593	0.0529
		(0.0550)		(0.0601)	(0.0598)
Discount rate (average)		0.0001		0.0003	0.0003
		(0.0009)		(0.0009)	(0.0009)
Risk preference (average)		-0.0004		-0.0008	-0.0008
		(0.0006)		(0.0006)	(0.0006)
Locus of control (average)		0.0091		0.0234	0.0215
		(0.0233)		(0.0247)	(0.0246)
Anxiety (average)		-0.0478**		-0.0300	-0.0292
		(0.0207)		(0.0220)	(0.0219)
Self-perception (average)		-0.0702***		-0.0265	-0.0292
		(0.0241)		(0.0258)	(0.0257)
Self-confidence (average)		-0.0333		-0.0214	-0.0197
		(0.0233)		(0.0249)	(0.0249)
Cognitive ability (average)		-0.0023		-0.0069	-0.0082
		(0.0086)		(0.0093)	(0.0092)
Male			0.0148	0.0172	0.0155
			(0.0149)	(0.0160)	(0.0159)
Age = 21			-0.1284***	-0.1279***	-0.1238***
			(0.0435)	(0.0436)	(0.0434)
Age = 22			-0.1668***	-0.1620***	-0.1581***
			(0.0412)	(0.0415)	(0.0414)
Age = 23			-0.1387***	-0.1337***	-0.1320***
			(0.0406)	(0.0412)	(0.0410)
Age = 24			-0.1456***	-0.1403***	-0.1385***
			(0.0407)	(0.0414)	(0.0412)
Age = 25			-0.1088***	-0.1010**	-0.0996**
			(0.0416)	(0.0427)	(0.0425)
Age = 26			-0.1181***	-0.1106**	-0.1122**
			(0.0434)	(0.0446)	(0.0445)
Age = 27			-0.1539***	-0.1441***	-0.1449***
			(0.0460)	(0.0475)	(0.0473)
Age = 28			-0.1626***	-0.1505***	-0.1464***
			(0.0521)	(0.0534)	(0.0533)
Age = 29			-0.1641***	-0.1515**	-0.1512**
			(0.0630)	(0.0638)	(0.0636)
Age = 30			-0.2640***	-0.2508***	-0.2512***
			(0.0692)	(0.0715)	(0.0712)
Educ father			-0.0007	-0.0018	-0.0024
			(0.0039)	(0.0042)	(0.0042)
Educ mother			-0.0054	-0.0041	-0.0038
			(0.0039)	(0.0042)	(0.0042)
Middle level sec. school			0.0687***	0.0702***	0.0637***
			(0.0229)	(0.0237)	(0.0241)
High level sec. school			0.0609**	0.0662**	0.0616**
			(0.0249)	(0.0262)	(0.0265)
Immigrant			0.0696***	0.0577**	0.0575**
			(0.0225)	(0.0249)	(0.0248)
Discount rate			-0.0001	-0.0002	-0.0005
			(0.0003)	(0.0003)	(0.0012)
Risk preference			0.0002	0.0003	0.0014**
			(0.0002)	(0.0002)	(0.0006)
Locus of control			-0.0096	-0.0125	-0.0405**
			(0.0081)	(0.0087)	(0.0198)
Anxiety			-0.0188***	-0.0151*	0.0085
			(0.0073)	(0.0078)	(0.0410)
Self-perception			-0.0467***	-0.0432***	-0.0338***
			(0.0086)	(0.0093)	(0.0128)
Self-confidence			-0.0140*	-0.0119	0.0025
			(0.0080)	(0.0086)	(0.0115)
Cognitive ability			-0.0020	-0.0010	0.0125
			(0.0034)	(0.0037)	(0.0101)
Discount rate squared					0.0000
					(0.0000)
Risk preference squared					-0.0000*
					(0.0000)
Locus of control squared					0.0098
					(0.0063)
Anxiety squared					-0.0028
					(0.0049)
Self-perception squared					0.0068
					(0.0055)
Self-confidence squared					0.0090*
					(0.0047)
Cognitive ability squared					-0.0019
					(0.0013)
Constant	0.2229***	0.5209**	0.3637***	0.6506***	0.5488**
	(0.0068)	(0.2136)	(0.0545)	(0.2315)	(0.2380)
Observations	4,191	4,191	4,191	4,191	4,191

Notes: Standard errors in parentheses,

*** p<0.01,

** p<0.05,

* p<0.1.

Data source: Supplement survey of the 2004 SIS wave. The dependent variable is a dummy variable (0 = does not prefer a different field of education in retrospect, 1 = prefers a different field of education in retrospect). Counseling is standardized at the school level as described in the data section. This variable is instrumented with the average amount of counseling by students of the same secondary school. Educ Father and Mother represent the highest level of education that the father or mother graduated from. “average” indicates that school averages have been calculated.

Taken at face value, the magnitude or our estimate implies that if counseling can be increased by a standard deviation, the average probability to prefer a different field will decrease by 9 percent (2 percentage points less than the original level of 22 percent).

Comparing the OLS and the IV estimate with the variance of counseling (5.882*5.882) and the variance of the prediction of counseling in the first stage (1.02*1.02), gives information about the nature of the bias in the OLS. To this extent, one could compare the ratio of the coefficients of the OLS and the IV to the ratio of the variances. If the IV would only correct for measurement error, the ratio of these two variances would equal the ratio of the IV estimate and the OLS estimate. In our case these ratios are not equal, which suggests that counseling is endogenous.

We consider these estimates to be large, especially since counseling is relatively inexpensive. The survey which was held among the counselors indicates that the average time of a counseling session is about 25 minutes per student and per meeting. In comparison, the costs which might be avoided are potentially large for the individual and from society’s point of view since some students who in retrospect would choose a different education may seek employment in a different line of work, others may continue working in the field they chose at the cost of a lower level of utility and/or productivity and others may re-enroll in a different education to correct their choice. In our sample, a low quality choice is correlated with continuing schooling (*p*-value 0.003). This is in line with results in [2] who, using a different sample, found the indicator of low quality of educational choice to be linked with a higher probability of re-enrollment in a different field of education at an adult age. For a year of adult education, calculations in [39] indicate a cost of at least €10,000 in individual foregone earnings. However, the need for re-schooling can only partly be addressed by counseling as it may be related to events which are impossible to foresee.

Table 7 separates the results for different subgroups. The point estimates of the effect of counseling are much larger for men (-0.048) than for women (-0.010), with the latter also insignificantly different from zero. Table E in S2 File shows the OLS regressions for the subgroups, overall indicating coefficient values which are insignificant and (or) very close to zero. This differs from earlier evidence, notably from [32] who find counseling to affect females, but not males, in the probability of attending college. A plausible explanation is that, as in many countries, there are strong gender patterns in career choices of low skilled people in the Netherlands, with males choosing between more heterogeneous career alternatives. This could explain the gender difference in estimates. There are many potential reasons for the different gender pattern reported in [32]. Their treatment includes mentoring and a cash grant, using observed college enrolments as outcome. As mentioned earlier, the individuals’ own assessment of their educational choice in our study is a different concept and the time-frame is 6–7 years. [32] suggest various mechanisms to explain the gender dissimilarity, and these may differ between our studies. Separate regression estimates for groups with different educational tracks at secondary level display large point estimates for individuals who attended the lowest secondary tracks (-0.042), while the effects for higher educational tracks are smaller and insignificant. The lowest track has the strongest focus on vocational education and students are traditionally recruited from relatively less affluent families. Restricting our sample to individuals with parents who have lower educational attainment than the median yields a significant effect of counseling (-0.038), whereas those whose parents have higher educational attainments than the median are associated with a modest estimate (-0.007, insignificant). The point estimate for individuals with immigrant background is high in absolute terms (-0.051) but there is a lack of precision in the estimates, as well as an insignificant first stage estimate (see Table F in S2 File), presumably due to the smaller sample (N = 413). Analyzing the effects for the separate subgroups simultaneously using interaction variables, yields significant differences between gender and between the low and the high secondary school tracks (see Table G in S2 File). When excluding immigrants, our results are similar to those reported in Table 6. In sum, a possible interpretation is that males from relatively low socioeconomic groups drive our overall significant results. This in line with the findings in [30].

**Table 7:** The effect of counseling on the quality of the educational choice by subgroups.

	(1)	(2)	(3)	(4)	(5)	(6)	(7)	(8)	(9)
	Women	Men	Low sec educ	Middle sec educ	High sec educ	Parents low	Parents high	Immigrants	Natives
Counseling	-0.0092	-0.0472**	-0.0404**	-0.0165	-0.0024	-0.0375**	-0.0083	-0.0605	-0.0197*
	(0.0132)	(0.0240)	(0.0194)	(0.0230)	(0.0176)	(0.0147)	(0.0248)	(0.1060)	(0.0117)
Full set of controls	Incl	Incl	Incl	Incl	Incl	Incl	Incl	Incl	Incl
Observations	2,650	1,541	1,080	1,653	1,813	1,277	1,492	413	3,778

Notes: Standard errors in parentheses,

** p<0.05,

* p<0.1.

Data source: Supplement survey of the 2004 SIS wave. The dependent variable is a dummy variable (0 = does not prefer a different field of education in retrospect, 1 = prefers a different field of education in retrospect). Counseling is standardized at the school level as described in the data section. This variable is instrumented with the average amount of counseling by students of the same secondary school. A full set of controls (see Table 6) is included in all regressions.

## Robustness Analyses

In this section, we present results from robustness checks to further check the validity of our IV strategy, complementing our discussions with respect to school endogeneity, and individual endogeneity or peer effects (see the empirical strategy Section above).

First, to assess if some unobserved school specific confounder(s) lead to an overestimation of the effects of counseling in our IV regressions, one may note that if school level factors tend to simultaneously influence counseling and quality of educational choice, one would also expect our observable school characteristics related to family background and pupil personality traits to have some impact on our IV estimates. However, the estimates in Table 6 remain remarkably stable as we add explanatory variables, providing little indication that school factors are driving the results.

Second, we have in total information on twelve guidance measures, of which three are strongly and positively correlated with the counseling indicator (significant at a .01 level; these were mentioned in the empirical strategy Section above). Results from IV regressions using other measures of guidance yield no statistically significant estimate on the quality of educational choice. See Table H in S2 File for the results. These results are also insignificant for our subgroups. Exceptions are that “people came to talk about their professions” is significant at the 10% level for those with higher educated parents. And “how often did you go to an information day” is significant at the 5% level for those from the higher track and at the 10% level for immigrants. The other measures of guidance are “Been to educational choice meeting in Utrecht,” “School has subscription to magazine about educational choice,” “Test for educational or professional choice,” “Extended documentation about educations and professions at school,” “I have had personal conversations with a mentor,” “I have spoken with friends about the educational choice,” “I have spoken with my parents about the educational choice,” “I made contact with people working or studying in the fields I thought were interesting,” and “I or my parents contacted a professional educational choice agency”.

Thus, a potential confounding factor must not only correlate with school counseling practices and students’ quality of educational choice, but also *not* correlate with any of the eleven other measures of guidance at the school level. This is in addition to not correlating with the observable school averages of parents’ social background, education and immigrant status, with school averages of pupils’ IQ, levels of anxiety and confidence as well as our four other personality traits.

Third, it might appear reasonable to include school fixed effects in our IV framework, either as explanatory variables (included in both the first and the second stage) or as an additional set of (567) instrumental variables (only included in the first stage), as it would explicitly control for school endogeneity. Note however that the first stage predictive power is then enhanced by the counseling incidence of the individuals themselves. This implies an obvious risk of over-identification which leads us back to the original endogeneity problem. As expected, running this estimation produces a coefficient estimate close to the OLS parameter (-.0025, *p*-value .078). Including *f*_*s*_ as additional covariate, i.e. also included in the second stage regression, yields similar results. Overall, our analyses indicate that unobserved factors at the school level can only account for modest bias, demonstrating support for our key assumption; that the variation in our original IV (*S*_*jt*_) comes from the counselor and not from the school. When excluding the school fixed effects, *S*_*jt*_ provides a continuous measure of the probability of seeing a counselor which is not flawed by the endogeneity of the individuals’ own decisions.

Another concern may be that, because of the varying size of the samples per school, measurement errors make the first stage heteroscedastic with respect to the number of students per school. This is foremost a problem in case the number of students per school responding to our survey is systematically related to the school counseling policy. We address this by interacting our IV with the number of people per school (in line with [40], employing three different specifications where we (1) interact the IV with the number of observations from the school (*N*_*sch*_), (2) interact the IV with above and below median of *N*_*sch*_ (10), and (3) interact the IV with quartiles of *N*_*sch*_ (5, 10 and 15). Table 8 shows that the effects remain similar.

**Table 8:** The effect of counseling on the quality of the educational choice taking into account potential heterogeneity with respect to the size of the school.

	(1)	(2)	(3)	(4)	(5)	(6)
	Linear	Linear	Median	Median	Quartiles	Quartiles
	Incl same cohort	Not incl samecohort	Incl same cohort	Not incl same cohort	Incl same cohort	Not incl same cohort
Counseling	-0.0190*	-0.0222*	-0.0228*	-0.0253**	-0.0177*	-0.0212*
	(0.0107)	(0.0122)	(0.0117)	(0.0127)	(0.0105)	(0.0114)
Full set of controls	Incl	Incl	Incl	Incl	Incl	Incl
Observations	4,191	4,165	4,191	4,165	4,191	4,165

Notes: Standard errors in parentheses,

** p<0.05,

* p<0.1.

Data source: Supplement survey of the 2004 SIS wave. The dependent variable is a dummy variable (0 = does not prefer a different field of education in retrospect, 1 = prefers a different field of education in retrospect). Counseling is standardized at the school level as described in the data section. The table presents 3 variants of 2 specifications of the instrument. In the specification “Incl same cohort” the counseling variable is instrumented with the average amount of counseling by students from the same secondary school. In the specification “Not incl same cohort” the variable is instrumented with the average amount of counseling by students from the same secondary school who did not graduate in the same year as the individual. In the Linear variant the instrument is replaced by the instrument, a variable indicating the number of people in a school, and the interaction between these two variables. In the Median variant, the instrument is replaced by the instrument, a dummy variable which has the value 1 if the number of individuals in the school is larger than 10 (the median), and the interaction between these two variables. In the Quartiles variant, the instrument is replaced by the instrument, 3 dummy variables of which the first has value 1 if the number of students in the school was between 5 (the first quartile) and 10, the second has a value 1 if the number was between 10 and 15 (the third quartile) and the third has value 1 if the number was more than 15, and interactions between the instrument and these dummy variables. A full set of controls (see Table 6) is included in all regressions.

Fourth, the IV may pick up peer effects between the students. However, each school is represented by relatively small samples of individuals who responded to the Internet survey, arguably making it unlikely in the first place that the students know each other or affect each other’s answers (see the empirical strategy Section). To further address this concern, we redefine our IV into the average counseling of individuals who graduated from the same secondary school but not in the same year as the individual. The results, reported in Table 9, show that the effect of counseling on educational choice quality remains similar and that this also holds for the subgroups. There is only a minor change in the first stage coefficient of our IV (from .5651 to .5162), further supporting the hypothesis that peer effects do not drive the estimates.

**Table 9:** The effect of counseling on the quality of the educational choice using the instrument which excludes students from the same cohort.

	(1)	(2)	(3)	(4)	(5)	(6)	(7)	(8)	(9)	(10)
	Full sample	Women	Men	Low sec educ	Middle sec educ	High sec educ	Parents low	Parents high	Immigrants	Natives
Counseling	-0.0244*	-0.0142	-0.0413*	-0.0343	-0.0157	-0.0120	-0.0401**	-0.0105	-0.0366	-0.0228*
	(0.0130)	(0.0158)	(0.0223)	(0.0231)	(0.0233)	(0.0196)	(0.0175)	(0.0252)	(0.1246)	(0.0130)
Full set of controls	Incl	Incl	Incl	Incl	Incl	Incl	Incl	Incl	Incl	Incl
Observations	4,165	2,635	1,530	1,072	1,649	1,798	1,272	1,478	408	3,757

Notes: Standard errors in parentheses,

** p<0.05,

* p<0.1.

Data source: Supplement survey of the 2004 SIS wave. The dependent variable is a dummy variable (0 = does not prefer a different field of education in retrospect, 1 = prefers a different field of education in retrospect). Counseling is standardized at the school level as described in the data section. This variable is instrumented with the average amount of counseling by students from the same secondary school who did not graduate in the same year as the individual. A full set of controls (see Table 6) is included in all regressions.

## Mechanisms

Given that counselors affect the quality of educational choice, we next consider if data may assist us to disentangle some of the underlying mechanisms. In the additional survey of the counselors, there were questions on the topics they discussed with the students during the individual counseling sessions. The answer categories included the awareness of the students’ motivation and competences, information about the courses given in secondary school, information about the courses given in tertiary education, and information about the labor market, including knowledge about the labor market, information about apprenticeships and insights in professions (answers were given on a scale from 1. Never– 5. Very often). We find our IV to be significantly related with conversations about the awareness of the students’ motivation and competences but not with the other answer categories. These correlations may suggest that the counseling variable we use as an IV primarily reflects actions addressing the individual’s uncertainty about his/her own utility function (future preferences), rather than information about objective measures such as wages and/or employment probabilities. A caveat is that we have no information on whether counselors approach individuals differently with respect to e.g. social background factors or ethnicity.

To analyze this issue further, we use our IV approach to see if counseling affects employment status (self-reported, 78.0 percent are employed). If employment probabilities consist of a permanent part and transitory shocks, counselors are in theory able to provide information to students about the permanent part. Our IV estimates are statistically insignificant throughout our subsamples (full sample *p*-value of .605), consistent with the idea that our LATE estimates of counseling are not primarily driven by information on employment probabilities. Note that within this framework, using employment incidence (or wage levels) as the outcome of interest is potentially misleading. Employment following acceptance of a low wage-offer may signal a low quality of educational choice. Wage levels may in addition carry little information when measured only 18 months after graduation. The result is in line with [11], who report that non-monetary aspects are important for the choice of major at French universities. An obvious reservation is that employment status here is only measured 18 months after graduation, with differences perhaps emerging later on. Future studies with longer time frames will be necessary to work out these mechanisms more precisely.

## Conclusions

In this paper, we present evidence that the quality of the educational choice is improved by study counseling. Our results indicate that visiting a study counselor decreases the average probability to prefer a different educational field by 2 percentage points (from a baseline of 22 percent), corresponding to a 9 percent decrease. The groups which we would expect to have the least information at the outset, students with low educated parents, appear to have the largest marginal effects of added information through counseling. Our main contribution is to have provided empirical support for the hypothesis that counseling matters for the quality of educational choice. This complements in three ways the large number of studies which report that information matters for educational choice (for references, see introduction). First, our treatment variable is a policy tool which exists in many countries. Second, our results are obtained despite a considerably longer time-span than previous studies as outcomes are assessed after graduation, encompassing individuals’ actual (rather than expected) experiences of their educational choice and initial labor market careers. Third, our outcome variable considers the individuals’ own assessments which means we take into account the weights attached to various aspects of the chosen career path. One may also note that our result is in line with evaluations of randomized job-search counseling, which indicate beneficial effects on labor market outcomes of improving information and/or motivation through individual meetings with professionals [41–44].

Concerning the interpretation of the quantitative effect, we would advocate caution since the frequency and the quality of counseling likely correlate. This means that our results partly reflect the impact of high quality counseling rather than the average amount of counseling. More research is needed to better understand the anatomy of uncertainty. We do not know if the main uncertainty concerns information on wages or earnings in different sectors, or whether it is a question of uncertainty about the individual’s own (future) utility function. As we tentatively address this issue, we cannot reject that uncertainty about own utility is as important as uncertainty about wages and/or employment prospects.

## Supporting Information

S1 File The Dutch educational system. (DOCX)

S2 File Additional tables. (DOCX)

S3 File Questions used to measure personality and economic preference parameters. (DOCX)

## References

[1] Murnane RJ. (2013). U.S. High School Graduation Rates: Patterns and Explanations. Journal of Economic Literature 51(2), 370–422.

[2] Borghans L, Golsteyn BHH. (2007). Skill Transferability, Regret and Mobility. Applied Economics 39(13), 1663–1677.

[3] Levhari D, Weiss Y. (1974). The Effect of Risk on the Investment in Human Capital. American Economic Review 64, 950–963.

[4] Olson L, White H, Shefrin HM. (1979). Optimal Investment in Schooling When Incomes Are Risky. Journal of Political Economy 87(3), 522–539.

[5] Kodde D. (1986). Uncertainty and the Demand for Education. The Review of Economics and Statistics 68(3), 460–467.

[6] Manski CF (2004). Measuring Expectations. Econometrica 72(5), 1329–1376.

[7] Dominitz J, Manski CF. (1996). Eliciting Student Expectations of the Returns to Schooling. Journal of Human Resources 31(1), 1–26.

[8] Betts J. (1996). What do Students Know about Wages? Evidence from a Survey of Undergraduates. Journal of Human Resources 31(1), 27–56.

[9] Kauffman KM. (2009). Understanding the Income Gradient in College Attendance in Mexico: The Role of Heterogeneity in Expected Returns to College. Mimeo, Stanford University.

[10] Arcidiacono P, Hotz J, Kang S. (2012). Modeling College Major Choice Using Elicited Measures of Expectations and Counterfactuals. Journal of Econometrics 166(1), 3–16.

[11] Beffy M, Fougère D, Maurel A. (2012). Choosing the Field of Study in Postsecondary Education: Do Expected Earnings Matter? The Review of Economics and Statistics 94(1), 334–347.

[12] Bettinger EP, Long BT, Oreopoulos P, Sanbonmatsu L. (2012). The Role of Application Assistance and Information in College Decisions: Results from the H&R Block FAFSA Experiment. Quarterly Journal of Economics 127(3), 1205–1242.

[13] Dinkelman T, Martínez C. (2011). Investing in Schooling in Chile: The Role of Information About Financial Aid for Higher Education. CEPR DP No. 8375.

[14] Høst A, Jensen VM, Nielsen LP. (2012). Increasing the Admission Rate to Secondary School: The Case of Primary School Student Career Guidance. Mimeo Copenhagen: The Danish national Centre for Social Research.

[15] Jensen R. (2010). The (Perceived) Returns to Education and the Demand for Schooling. Quarterly Journal of Economics 125(2), 515–548.

[16] Nguyen T. (2008). Information, Role Models and Perceived Returns to Education: Experimental Evidence from Madagascar. Mimeo MIT.

[17] Oreopoulos P, Dunn R. (2013). Providing Information and Increasing Knowledge About Post Secondary Education: Evidence from a Randomized Field Experiment. Scandinavian Journal of Economics 115(1), 3–26.

[18] Papay JP, Murnane RJ, Willett JB. (2011). How Performance Information Affects Human-Capital Investment Decisions: The Impact of Test-Score Labels on Educational Outcomes. NBER Working Paper No. 17120.

[19] Stinebrickner TR, Stinebrickner R. (2011). Math or Science? Using Longitudinal Expectations Data to Examine the Process of Choosing a College Major. NBER working paper 16869.

[20] Stinebrickner TR, Stinebrickner R. (2012). Learning about Academic Ability and the College Dropout Decision. Journal of Labor Economics 30(4), 707–748.

[21] Zafar B. (2011). How Do College Students Form Expectations? Journal of Labor Economics 29(2), 301–348.

[22] Akerlof GA, Kranton RE(2000), Economics and Identity. Quarterly Journal of Economics 115(3), 715–753.

[23] Akerlof GA, Kranton RE (2002), Identity and Schooling: Some Lessons for the Economics of Education. Journal of Economic Literature 40, 1167–1201.

[24] Favara M. (2011). The Cost of Acting “Girly”: Gender Stereotypes and Educational Choices. Paper presented at the EALE conference, Cyprus September 2011.

[25] Humlum M, Kleinjans K, Nielsen H. (2012). An Economic Analysis of identity and Career Choice. Economic Inquiry 50(1), 39–61.

[26] Bandura A. (1977). Self-efficacy: Toward a Unifying Theory of Behavioral Change. Psychological Review 84(2), 191–215. 847061 10.1037//0033-295x.84.2.191

[27] Whinston S, Sexton TL, Lasoff DL. (1998). Career-Intervention Outcome: a Replication and Extension of Oliver and Spokane. Journal of Counseling Psychology 45(2), 150–165.

[28] Kraus LJ, Hughey KF. (1999). The Impact of an Intervention on Career Decision-Making Self-Efficacy and Career Indecision. Professional School Counseling, 2(5), 384–390.

[29] Jurgens JC. (2000). The Undecided Student: Effects of Combining Levels of Treatment Parameters on Career Certainty, Career Indecision, and Client Satisfaction. Career Development Quarterly 48, 237–250.

[30] Cunha J, Miller T. (2009). Information and the Decision to Attend College: Evidence from the Texas GO Center Project. Unpublished manuscript.

[31] Avery C. (2010). The Effects of College Counseling on High-Achieving, Low-Income Students. NBER Working paper 16359.

[32] Carrell S, Sacerdote B. (2013). Late Interventions Matter Too: The Case of College Coaching in New Hampshire. NBER working paper, W19031.

[33] Borghans L, Duckworth A, Heckman J, ter Weel B. (2008). The Economics and Psychology of Personality Traits. Journal of Human Resources 43, 972–1059.

[34] Roberts B, DelVecchio W. (2000). The Rank-Order Consistency of Personality Traits from Childhood to Old Age: A Quantitative Review of Longitudinal Studies. Psychological Bulletin 126(1), 3–25. 10668348 10.1037/0033-2909.126.1.3

[35] Imbens G, Angrist J. (1994). Identification and Estimation of Local Average Treatment Effects. Econometrica 62(2), 467–475.

[36] Angrist J. and Krueger A. (2001). Instrumental Variables and the Search for Identification: From Supply and Demand to Natural Experiments. Journal of Economic Perspectives 15(4), 69–85.

[37] Moffitt R. (2004). Remarks on the Analysis of Causal Relationships in Population Research. Working Paper, Johns Hopkins University (published version from 2003; “Causal Analysis in Population Research: An Economist’s Perspective”, Population and Development Review 29(3), 448–458.

[38] Manski CF. (1993). Identification of Endogenous Social Effects: The Reflection Problem. The Review of Economic Studies 60(3), 531–542.

[39] Stenberg A. (2011). Using Longitudinal Data to Evaluate Publicly Provided Formal Education for Low-skilled. Economics of Education Review 30(6), 1262–1280.

[40] Card D. (1995). Using Geographic Variation in College Proximity to Estimate the Return to Schooling, NBER Working Paper No. 4483.

[41] Behaghel L, Crépon B, Gurgand M. (2012). Private and Public Provision of Counseling to Job-Seekers: Evidence from a Large Controlled Experiment. IZA Discussion Paper No. 6518.

[42] Crépon B, Dejemeppe M, Gurgand M. (2005). Counseling the Unemployed: Does it Lower Unemployment Duration and Recurrence? IZA Discussion Paper 1796.

[43] Hainmüller J, Hofmann B, Krug G, Wolf K. (2009). Do More Placement Officers Lead to Lower Unemployment? IAB Discussion Paper, 13.

[44] Pedersen JM, Rosholm M, Svarer M. (2012). Experimental Evidence on the Effects of Early Meetings and Activation. Paper presented at ESPE, Bonn, June 2012.

