# Supplementary material for: Does Expert Advice Improve Educational Choice?
Source: PLoS One. 2015 Dec 21;10(12):e0145378. doi: 10.1371/journal.pone.0145378 (PMC4686924; doi:10.1371/journal.pone.0145378)
Supplement: S1 File — (DOCX) [file pone.0145378.s001.docx]

**S1. The Dutch educational system**

This appendix gives more information about the Dutch educational system. For more detailed information on the Dutch educational system we refer to: <https://en.wikipedia.org/wiki/Education_in_the_Netherlands> (retrieved 8 November 2015)

The Dutch educational system consists of primary, secondary, and tertiary levels.

Pupils attend primary education at the age of 4-12. Specific features of primary education are:

- Courses are taught at the same level to all students;
- In the last year of primary school (age 12), students take the CITO Achievement test and receive an advice from elementary school teachers to determine whether they will attend VMBO, HAVO, or VWO secondary school.

There are three levels of secondary education:

- VMBO (4 years), preparing for vocational education
- HAVO (5 years), preparing for professional college
- VWO (6 years), preparing for university

A specific feature of secondary education is that within each level courses are taught at the same level to all students.

There are three levels of tertiary education:

- MBO: vocational education
- HBO: professional college
- WO: university

A specific feature is again that within each level, courses taught at same level to all students.
