# Supplementary material for: Does Expert Advice Improve Educational Choice?
Source: PLoS One. 2015 Dec 21;10(12):e0145378. doi: 10.1371/journal.pone.0145378 (PMC4686924; doi:10.1371/journal.pone.0145378)
Supplement: S2 File — (DOCX) [file pone.0145378.s002.docx]

**S2. Additional tables**

**Table A. Quality of educational choice in various countries.**

|  | Average  answer | N | St. dev. | Percentage preferring a different field  (answering 4 not likely or 5 not likely at all) |
| --- | --- | --- | --- | --- |
| Italy | 2.3 | 2991 | 1.3 | 23.7 |
| Spain | 2.2 | 3001 | 1.3 | 20.4 |
| France | 2.2 | 3011 | 1.3 | 21.0 |
| Austria | 2.1 | 2286 | 1.3 | 19.5 |
| Germany | 2.2 | 3464 | 1.3 | 20.5 |
| The Netherlands | 2.3 | 3059 | 1.2 | 18.1 |
| UK | 2.4 | 3351 | 1.4 | 24.8 |
| Finland | 2.1 | 2648 | 1.2 | 17.5 |
| Sweden | 2.2 | 2606 | 1.3 | 20.0 |
| Norway | 2.1 | 3280 | 1.2 | 15.1 |
| Czech Republic | 2.3 | 3076 | 1.2 | 24.4 |
| Japan | 2.7 | 3287 | 1.3 | 30.7 |
| Total | 2.3 | 36058 | 1.3 | 21.4 |

Source: CHEERS, 1998. Graduates from higher vocational education and university were approached 3 years after graduation with the question ‘Looking back, if you were free to choose again, would you choose the same study program?’. The answers are scaled from (1) ‘very probable’ to (5) ‘not likely at all’.

**Table B. Analyses of data attrition.**

|  | Sample 1 | Sample 2 |
| --- | --- | --- |
|  | Respondents first wave | Respondents second wave after selecting usable cases |
| % Male | 44.1 | 36.8 |
| Average age | 24.1 | 24.2 |
| % Low level tertiary (MBO) | 25.5 | 16.0 |
| % Middle level tertiary (HBO | 47.8 | 54.0 |
| % High level tertiary (University) | 26.6 | 30.0 |
| % Immigrants | 12.2 | 9.9 |
| % Low quality educational choice | 22.9 | 22.0 |
| N | 27,929 | 4,191 |

Data source: 2004 SIS and Supplement survey of the 2004 SIS. In both data we selected MBO, HBO, and University respondents between the ages of 20 and 30.

**Table C. Averages of individual characteristics by gender and the incidence of seeing a counselor.**

|  | Women | |  | Men | |  |
| --- | --- | --- | --- | --- | --- | --- |
|  | No counseling | Counseling |  | No counseling | Counseling |  |
| Age | 23.95 | 23.95 |  | 24.42 | 24.55 |  |
| Education father | 4.50 | 4.57 |  | 4.52 | 4.43 |  |
| Education mother | 3.64 | 3.65 |  | 3.67 | 3.58 |  |
| % low level secondary school | 0.32 | 0.23 | *** | 0.30 | 0.24 | *** |
| % middle level secondary school | 0.37 | 0.41 | *** | 0.35 | 0.41 | *** |
| % high level secondary school | 0.39 | 0.44 | *** | 0.44 | 0.46 | *** |
| % immigrant | 0.11 | 0.11 |  | 0.10 | 0.08 |  |
| Discount rate | 0.09 | 0.03 |  | -0.07 | -0.08 |  |
| Risk aversion | 0.14 | 0.10 |  | -0.23 | -0.18 |  |
| Locus of control | -0.05 | -0.05 |  | 0.08 | 0.09 |  |
| Anxiety | 0.09 | 0.11 |  | -0.19 | -0.17 |  |
| Self-perception | -0.03 | -0.05 |  | 0.06 | 0.08 |  |
| Self-confidence | -0.07 | -0.03 |  | 0.11 | 0.06 |  |
| Cognitive ability | -0.32 | -0.22 | ** | 0.47 | 0.41 |  |

Notes: Stars indicate whether the means differ significantly: *** p<0.01, ** p<0.05, * p<0.1. In this table, discount rate, risk aversion, locus of control, anxiety, self-perception, self-confidence and cognitive ability are standardized with mean zero and standard deviation one. Education father and mother are measured on a 7 point scale ranging from 1 primary education to 7 university.

**Table D. OLS regressions of seeing a counselor and quality of educational choice on set of variables.**

|  | (1) | (2) | (3) | (4) |
| --- | --- | --- | --- | --- |
|  | Seeing a  Counselor | Seeing a  Counselor | Prefers a  different field | Prefers a  different field |
| Male | 0.0076 | 0.0073 | 0.0126 | 0.0110 |
|  | (0.0160) | (0.0160) | (0.0143) | (0.0143) |
| Age=21 | 0.0007 | 0.0005 | -0.1286*** | -0.1243*** |
|  | (0.0466) | (0.0466) | (0.0417) | (0.0417) |
| Age=22 | -0.0010 | -0.0066 | -0.1666*** | -0.1602*** |
|  | (0.0442) | (0.0441) | (0.0395) | (0.0395) |
| Age=23 | 0.0041 | 0.0003 | -0.1399*** | -0.1360*** |
|  | (0.0435) | (0.0435) | (0.0390) | (0.0389) |
| Age=24 | -0.0263 | -0.0303 | -0.1380*** | -0.1336*** |
|  | (0.0435) | (0.0435) | (0.0389) | (0.0389) |
| Age=25 | -0.0118 | -0.0113 | -0.1054*** | -0.1023** |
|  | (0.0446) | (0.0445) | (0.0399) | (0.0398) |
| Age=26 | -0.0171 | -0.0148 | -0.1132*** | -0.1135*** |
|  | (0.0464) | (0.0464) | (0.0415) | (0.0415) |
| Age=27 | -0.0078 | -0.0103 | -0.1517*** | -0.1494*** |
|  | (0.0493) | (0.0492) | (0.0441) | (0.0440) |
| Age=28 | -0.0657 | -0.0716 | -0.1437*** | -0.1361*** |
|  | (0.0550) | (0.0550) | (0.0492) | (0.0492) |
| Age=29 | -0.1387** | -0.1397** | -0.1241** | -0.1214** |
|  | (0.0643) | (0.0642) | (0.0576) | (0.0575) |
| Age=30 | 0.0230 | 0.0226 | -0.2706*** | -0.2671*** |
|  | (0.0741) | (0.0740) | (0.0664) | (0.0662) |
| Educ father | -0.0007 | -0.0002 | -0.0005 | -0.0011 |
|  | (0.0042) | (0.0042) | (0.0038) | (0.0038) |
| Educ mother | -0.0018 | -0.0023 | -0.0049 | -0.0043 |
|  | (0.0042) | (0.0042) | (0.0037) | (0.0037) |
| Middle level secondary school | 0.0987*** | 0.1007*** | 0.0402** | 0.0347** |
|  | (0.0197) | (0.0198) | (0.0176) | (0.0177) |
| High level secondary school | 0.1033*** | 0.1038*** | 0.0311 | 0.0280 |
|  | (0.0218) | (0.0218) | (0.0195) | (0.0195) |
| Immigrant | -0.0192 | -0.0175 | 0.0751*** | 0.0733*** |
|  | (0.0240) | (0.0240) | (0.0215) | (0.0215) |
| Discount rate | -0.0003 | -0.0016 | -0.0000 | -0.0001 |
|  | (0.0003) | (0.0012) | (0.0003) | (0.0011) |
| Risk preference | -0.0000 | -0.0000 | 0.0002 | 0.0013** |
|  | (0.0002) | (0.0007) | (0.0002) | (0.0006) |
| Locus of control | -0.0024 | 0.0003 | -0.0089 | -0.0369** |
|  | (0.0087) | (0.0210) | (0.0078) | (0.0188) |
| Anxiety | 0.0057 | 0.1086*** | -0.0204*** | -0.0242 |
|  | (0.0078) | (0.0403) | (0.0069) | (0.0361) |
| Self-perception | -0.0031 | -0.0174 | -0.0458*** | -0.0320*** |
|  | (0.0092) | (0.0128) | (0.0082) | (0.0115) |
| Self-confidence | 0.0037 | -0.0084 | -0.0150* | 0.0031 |
|  | (0.0086) | (0.0119) | (0.0077) | (0.0107) |
| Cognitive ability | -0.0016 | -0.0005 | -0.0016 | 0.0107 |
|  | (0.0037) | (0.0107) | (0.0033) | (0.0095) |
| Discount rate squared |  | 0.0000 |  | 0.0000 |
|  |  | (0.0000) |  | (0.0000) |
| Risk preference squared |  | 0.0000 |  | -0.0000* |
|  |  | (0.0000) |  | (0.0000) |
| Locus of control squared |  | -0.0004 |  | 0.0095 |
|  |  | (0.0067) |  | (0.0060) |
| Anxiety squared |  | -0.0127*** |  | 0.0006 |
|  |  | (0.0049) |  | (0.0044) |
| Self-perception squared |  | -0.0099* |  | 0.0101** |
|  |  | (0.0057) |  | (0.0051) |
| Self-confidence squared |  | -0.0069 |  | 0.0110** |
|  |  | (0.0049) |  | (0.0044) |
| Cognitive ability squared |  | -0.0002 |  | -0.0018 |
|  |  | (0.0014) |  | (0.0013) |
| Constant | 0.6326*** | 0.4635*** | 0.3799*** | 0.3668*** |
|  | (0.0578) | (0.0972) | (0.0518) | (0.0869) |
| Observations | 4,191 | 4,191 | 4,191 | 4,191 |
| R-squared | 0.012 | 0.017 | 0.028 | 0.035 |

Standard errors in parentheses, *** p<0.01, ** p<0.05, * p<0.1.

**Table E. OLS estimates of the relationship between study counseling and quality of the educational choice by subgroups.**

|  | (1) | (2) | (3) | (4) | (5) | (6) | (7) | (8) | (9) |
| --- | --- | --- | --- | --- | --- | --- | --- | --- | --- |
|  | Women | Men | Low  sec educ | Middle  sec educ | High  sec educ | Parents low | Parents high | Immigrants | Natives |
| Counseling | -0.0024* | -0.0005 | -0.0005 | -0.0021 | -0.0020 | -0.0063*** | 0.0009 | -0.0008 | -0.0016 |
|  | (0.0014) | (0.0018) | (0.0021) | (0.0018) | (0.0017) | (0.0020) | (0.0018) | (0.0038) | (0.0011) |
| Full set of controls | Incl | Incl | Incl | Incl | Incl | Incl | Incl | Incl | Incl |
| Observations | 2,650 | 1,541 | 1,080 | 1,653 | 1,813 | 1,277 | 1,492 | 413 | 3,778 |

Notes: Standard errors in parentheses, *** p<0.01, ** p<0.05, * p<0.1. Data source: Supplement survey of the 2004 SIS wave. The dependent variable is a dummy variable (0= does not prefer a different field of education in retrospect, 1= prefers a different field of education in retrospect). Counseling is standardized at the school level as described in the data section. A full set of controls (see table 6) is included in all regressions.

**Table F. First stage results: the effect of average amount of counseling by students of the same secondary school on individual’s counseling by subgroups.**

|  | (1) | (2) | (3) | (4) | (5) | (6) | (7) | (8) | (9) |
| --- | --- | --- | --- | --- | --- | --- | --- | --- | --- |
|  | Women | Men | Low  sec educ | Middle  sec educ | High  sec educ | Parents low | Parents high | Immigrants | Natives |
| Instrument | 2.9876*** | 2.5983*** | 3.5573*** | 2.2685*** | 2.7725*** | 4.2942*** | 2.0622*** | 1.2490 | 2.8864*** |
|  | (0.5613) | (0.7318) | (0.8824) | (0.7087) | (0.6842) | (0.8113) | (0.7513) | (1.3989) | (0.4706) |
| Full set of controls | Incl | Incl | Incl | Incl | Incl | Incl | Incl | Incl | Incl |
| Observations | 2,650 | 1,541 | 1,080 | 1,653 | 1,813 | 1,277 | 1,492 | 413 | 3,778 |
| R-squared | 0.035 | 0.049 | 0.066 | 0.031 | 0.039 | 0.071 | 0.038 | 0.099 | 0.033 |

Notes: Standard errors in parentheses, *** p<0.01, ** p<0.05, * p<0.1. Data source: Supplement survey of the 2004 SIS wave. The dependent variable “Counseling” is standardized at the school level as described in the data section. “Instrument” is the average amount of counseling by students of the same secondary school. A full set of controls (see table 6) is included in all regressions.

**Table G. The effect of counseling on the quality of the educational choice with interactions for subgroups.**

|  | (1) | (2) | (3) | (4) | (5) |
| --- | --- | --- | --- | --- | --- |
|  | Prefers a different field | Prefers a different field | Prefers a different field | Prefers a different field | Prefers a different field |
|  |  |  |  |  |  |
| Counseling | -0.0219* | -0.0110 | -0.0547** | -0.0525** | -0.1145** |
|  | (0.0118) | (0.0139) | (0.0235) | (0.0241) | (0.0461) |
| Counseling*Male |  | -0.0302 | -0.0450* | -0.0461* | -0.0573* |
|  |  | (0.0218) | (0.0256) | (0.0264) | (0.0305) |
| Counseling*Middle level secondary school |  |  | 0.0533 | 0.0539 | 0.0618 |
|  |  |  | (0.0352) | (0.0354) | (0.0417) |
| Counseling*High level secondary school |  |  | 0.0786** | 0.0781** | 0.0874** |
|  |  |  | (0.0321) | (0.0324) | (0.0371) |
| Counseling*Immigrant |  |  |  | -0.0236 | -0.0038 |
|  |  |  |  | (0.0645) | (0.0731) |
| Counseling*Educ father |  |  |  |  | 0.0035 |
|  |  |  |  |  | (0.0091) |
| Counseling*Educ mother |  |  |  |  | 0.0133 |
|  |  |  |  |  | (0.0106) |
| Full set of controls | Incl | Incl | Incl | Incl | Incl |
| Observations | 4,191 | 4,191 | 4,191 | 4,191 | 4,191 |

Notes: Standard errors in parentheses, *** p<0.01, ** p<0.05, * p<0.1. Data source: Supplement survey of the 2004 SIS wave. The dependent variable is a dummy variable (0= does not prefer a different field of education in retrospect, 1= prefers a different field of education in retrospect). Counseling is standardized at the school level as described in the data section. This variable is instrumented with the average amount of counseling by students of the same secondary school. The interaction variables are instrumented by the interaction of the instrument and the sub group variable, e.g. Counseling*Male is instrumented with the Instrument*Male, Counseling*Middle level secondary school is instrumented with the Instrument*Middle level secondary school, etc. A full set of controls (see table 6) is included in all regressions.

**Table H. The effect of other measures of guidance on the quality of the educational choice.**

|  | Coefficient | t-value |
| --- | --- | --- |
| We had lessons about educational choice in school | -0.341 | -1.062 |
| People came to talk about their professions | 0.031 | 0.403 |
| I have had personal conversations with a mentor | 0.121 | 0.448 |
| I have spoken with friends about the educational choice | -0.861 | -0.652 |
| I have spoken with my parents about the educational choice | 0.401 | 0.154 |
| I made contact with people working or studying in fields I thought were interesting | -0.301 | -0.528 |
| We had an educational choice test in school | -0.093 | -0.528 |
| We had a lot of documentation about educational choice in school | -0.155 | -1.053 |
| I got an educational choice magazine | -0.133 | -0.194 |
| I went to a meeting about educational choice in Utrecht | 0.076 | 0.863 |
| I or my parents contacted a professional educational choice agency | -2.941 | -0.526 |
| How often did you go to an information day? | -0.097 | -1.550 |

Notes: Data source: Supplement survey of the 2004 SIS wave. Each row shows the result of a separate IV regression. The dependent variable is a dummy variable (0= does not prefer a different field of education in retrospect, 1= prefers a different field of education in retrospect). The variable indicated in the row is the is standardized at the school level as described in the data section. This variable is instrumented with the average of that variable for students in the same secondary school. A full set of controls (see table 6) is included in all regressions.
