# Supplementary material for: Does Expert Advice Improve Educational Choice?
Source: PLoS One. 2015 Dec 21;10(12):e0145378. doi: 10.1371/journal.pone.0145378 (PMC4686924; doi:10.1371/journal.pone.0145378)
Supplement: S3 File — (DOCX) [file pone.0145378.s003.docx]

**S3. Questions used to measure personality and economic preference parameters**

**Time preference**

We used the average answer of 3 bundles of questions.

Question 1 reads as follows:

We now offer you a number of choices. Please indicate which alternative you would choose. It is important to know that we are not searching for the right answer. This answer does not exist. We are merely interested in your choices.

a O 800 euros now or O 1200 euros in one year

This question is repeated twice where the amounts change depending on the choices made. So:

b O X euros now or O Y euros in one year

c O X euros now or O Y euros in one year

| a | B | c | Resulting discount rate |
| --- | --- | --- | --- |
| X = 800  Y = 1200 | If X:  X = 800  Y = 1400 | If X:  X = 800  Y = 1500 | If X:  DR2=94 |
|  |  |  | If Y:  DR2=81 |
|  |  | If Y:  X = 800  Y = 1300 | If X:  DR2=69 |
|  |  |  | If Y:  DR2=56 |
|  | If Y:  X= 800  Y = 1000 | If X:  X = 800  Y = 1100 | If X:  DR2=44 |
|  |  |  | If Y:  DR2=31 |
|  |  | If Y:  X = 800  Y = 900 | If X:  DR2=19 |
|  |  |  | If Y:  DR2=6 |

Question 2 reads as follows:

What would you choose:

a O 800 euros now or O 4000 euros in four years

This question is repeated twice where the amounts change depending on the choices made. So:

b O X euros now or O Y euros in four years

c O X euros now or O Y euros in four years

| a | b | c | Resulting discount rate |
| --- | --- | --- | --- |
| X = 1000  Y = 4000 | If X:  X = 1000  Y = 6500 | If X:  X = 1000  Y = 8500 | If X:  DR3=75 |
|  |  |  | If Y:  DR3=65 |
|  |  | If Y:  X = 1000  Y = 5000 | If X:  DR3=55 |
|  |  |  | If Y:  DR3=45 |
|  | If Y:  X= 1000  Y = 2000 | If X:  X = 1000  Y = 3000 | If X:  DR3=37 |
|  |  |  | If Y:  DR3=25 |
|  |  | If Y:  X = 1000  Y = 1500 | If X:  DR3=15 |
|  |  |  | If Y:  DR3=5 |

Question 3 reads as follows:

What would you choose

a O 800 euros in one year or O 1200 euros in two years

This question is repeated twice where the amounts change depending on the choices made. So:

b O X euros in one year or O Y euros in two years

c O X euros in one year or O Y euros in two years

| A | b | c | Resulting discount rate |
| --- | --- | --- | --- |
| X = 800  Y = 1200 | If X:  X = 800  Y = 1400 | If X:  X = 800  Y = 1500 | If X:  DR4=94 |
|  |  |  | If Y:  DR4=81 |
|  |  | If Y:  X = 800  Y = 1300 | If X:  DR4=69 |
|  |  |  | If Y:  DR4=56 |
|  | If Y:  X= 800  Y = 1000 | If X:  X = 800  Y = 1100 | If X:  DR4=44 |
|  |  |  | If Y:  DR4=31 |
|  |  | If Y:  X = 800  Y = 900 | If X:  DR4=19 |
|  |  |  | If Y:  DR4=6 |

**Risk Preference**

We used the average answer to 2 bundles of questions

The first question reads:

Please indicate which alternative you would choose.

a O 800 euros or O 50% chance to get nothing and 50% chance to get 2000 euros

This question is repeated twice where the amounts change depending on the choices made. So:

b O X euros or O 50% chance to get nothing and 50% chance to get Y euros

c O X euros or O 50% chance to get nothing and 50% chance to get Y euros

| a | b | c | Resulting risk aversion |
| --- | --- | --- | --- |
| X = 800  Y = 2000 | If X:  X = 800  Y = 2400 | If X:  X = 800  Y = 2600 | If X:  RP1=68 |
|  |  |  | If Y:  RP1=56 |
|  |  | If Y:  X = 800  Y = 2200 | If X:  RP1=43 |
|  |  |  | If Y:  RP1=31 |
|  | If Y:  X= 800  Y = 1600 | If X:  X = 800  Y = 1800 | If X:  RP1=18 |
|  |  |  | If Y:  RP1=7 |
|  |  | If Y:  X = 800  Y = 1400 | If X:  RP1=-7 |
|  |  |  | If Y:  RP1=-18 |

Question 2 reads as follows:

What would you choose

a. O 100 euros of O 90% chance on nothing and 10% chance on 1500 euros

This question is repeated twice where the amounts change depending on the choices made. So:

b O X euros or O 90% chance to get nothing and 10% chance to get Y euros

c O X euros or O 90% chance to get nothing and 10% chance to get Y euros

| a | b | c | Resulting risk aversion |
| --- | --- | --- | --- |
| X = 100  Y = 1500 | If X:  X = 100  Y = 2000 | If X:  X = 100  Y = 2250 | If X:  RP2=136 |
|  |  |  | If Y:  RP2=112 |
|  |  | If Y:  X = 100  Y = 1750 | If X:  RP2=87 |
|  |  |  | If Y:  RP2=62 |
|  | If Y:  X= 100  Y = 1000 | If X:  X = 100  Y = 1250 | If X:  RP2=37 |
|  |  |  | If Y:  RP2=12 |
|  |  | If Y:  X = 100  Y = 750 | If X:  RP2=-12 |
|  |  |  | If Y:  RP2=-37 |

## Cognitive Reflection Test

Frederick, S. (2005). Cognitive reflection and decision making. *Journal of Economic Perspectives*. 19(4). 25-42.

Below there are 8 problems which differ in degree of difficulty. Try to answer as many questions as possible.

1. Together, a ball and a cap cost 1.10 Euros. The ball costs 1.00 Euros more than the cap. How much does the cap cost? ______ cents
2. If you toss a fair coin twice, how large is the chance that ‘Head’ comes up at least once?______ %
3. If 5 machines need 5 minutes to produce 5 things, how long do 100 machines need to make 100 things?______ minutes
4. Two cars are approaching each other in the same lane. Car A drives at a speed of 120 km/h. Car B at 60 km/h. How large is the distance between these two cars one minute before they collide? ______ Kilometers
5. In a lake there is a patch of lily pads. Every day the patch doubles in size. If it takes 48 days for the patch to cover the entire lake, how long would it take for the patch to cover half the lake? ______ days
6. If Timo drinks a bottle of water in 6 days and Esther takes 12 days to finish a bottle, how long does it take before they finish one bottle together? ______ dagen (answer=4)
7. If three salesmen can pack six toys in half an hour, how many salesmen would one need to pack 20 toys in one hour? ______ salesmen
8. At a match, Bart comes in at the 15^th^ place and at the 15^th^ last place. How many people participated at the match? ______ people

Answers: 5, 75, 5, 3, 47, 4, 5, 29.

**Personality traits**

We used answers to the following statements to measure personality traits.

Indicate how these statements relate to you on a scale 1: Totally disagree … 7: Totally agree

Locus of Control

- - Set backs are usually due to mistakes people make
  - Most people do not realize to what extent their life is determined by coincidences
  - Whether I reach targets that I have in my life is not a matter of luck

Anxiety

- - I often think about unpleasant events in the past
  - I often tend to check whether I did everything right
  - I think it is scary to go to places I have never been to

Self-Perception

- - My opinions about myself seem to change regularly
  - In general I have a clear idea about who and what I am
  - I often doubt about decisions because I do not know exactly what I want

Self-Confidence

- - I tend to think someone else is better than I am
  - I think I have enough reason to be proud of myself
  - The difference between who I am and what I want is large
